# Ceriantharia (Cnidaria) of the World: an annotated catalogue and key to species

**DOI:** 10.3897/zookeys.952.50617

**Published:** 2020-07-23

**Authors:** Sérgio N. Stampar, James D. Reimer, Maximiliano M. Maronna, Celine S. S. Lopes, Hellen Ceriello, Thais B. Santos, Fabián H. Acuña, André C. Morandini

**Affiliations:** 1 Universidade Estadual Paulista (UNESP), FCL/Assis, Laboratório de Evolução e Diversidade Aquática; LEDA, Departamento de Ciências Biológicas, Assis, Brazil; 2 University of The Ryukyus, Faculty of Science, Department of Biology, Chemistry, and Marine Science, MISE (Molecular Invertebrate Systematics and Ecology) Laboratory, Okinawa, Japan; 3 University of The Ryukyus, Tropical Biosphere Research Center, Okinawa, Japan; 4 Universidade de São Paulo (USP), Instituto de Biociências, São Paulo, SP, Brazil; 5 Universidade Estadual Paulista (UNESP), Departamento de Zoologia, Instituto de Biociências, Botucatu, SP, Brazil; 6 Instituto de Investigaciones Marinas y Costeras (Iimyc) CONICET; Facultad De Ciencias Exactas y Naturales Universidad Nacional de Mar Del Plata Funes 3250. 7600 Mar Del Plata, Argentina; 7 Estación Científica Coiba (Coiba-Aip), Clayton, Panamá, República de Panamá; 8 Universidade de São Paulo (USP), Centro de Biologia Marinha, São Sebastião, SP, Brazil

**Keywords:** Cnidaria, families, genera, identification key, tube-dwelling anemones

## Abstract

The diversity of Ceriantharia is known from studies formally describing species from the late 18^th^ Century onwards. However, no nomenclators including a list and discussion of all valid species have been produced since a list discussed by Carlgren in 1912. The present nomenclator presents a complete list of adult species of Ceriantharia of the World, including a discussion on each species. It includes the three families (Arachnactidae, Botrucnidiferidae, Cerianthidae) and the currently accepted 54 species based on their adult form. This study serves as a presentation of the “state-of-the-art” list of species of Ceriantharia, and includes a species identification key to support taxonomic identification. Additional in-depth species-by-species investigations for almost all cerianthid species is still needed, as the information available for most of these species is quite superficial.

## Introduction

**Figure 1.**
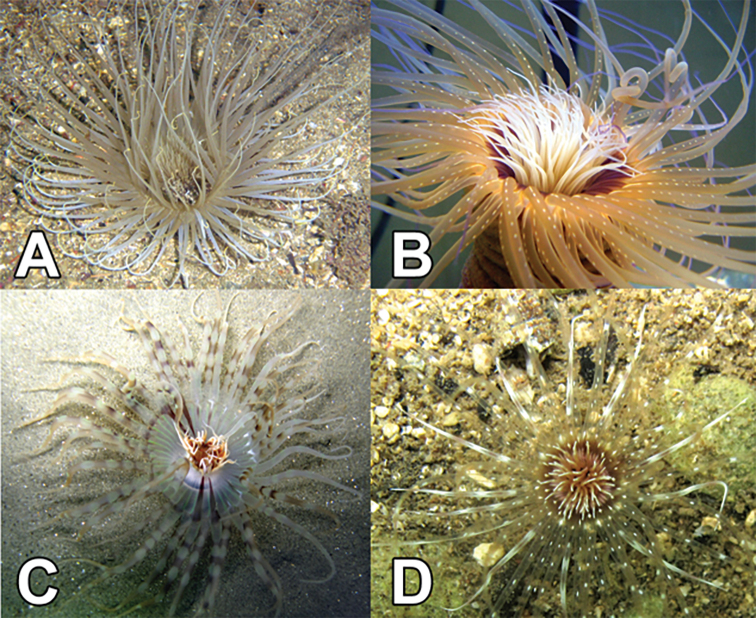
Examples of Ceriantharia: **A**Cerianthidae, *Ceriantheomorphe brasiliensis***B***Pachycerianthus schlenzae***C**Arachnactidae, *Isarachnanthus nocturnus***D**Botrucnidiferidae, *Botruanthus mexicanus* (Photograph Ricardo Gonzalez-Muñoz).

The subclass Ceriantharia Perrier, 1893 (Fig. 1), a group of anthozoan species commonly known as ‘tube anemones’, is characterized by the presence of two tentacle discs and a tube produced by filaments of a special kind of cnida, the ptychocyst (Daly et al. 2007; Stampar et al. 2016). This group of sediment-dwelling species is recognized as some taxa are common as pets in the aquarium industry (Stampar and Silveira 2006). On the other hand, the general taxonomy of the group is rather confusing, and the literature includes some incorrect definitions of characters for both species and genera (Torelli 1961; Stampar et al. 2012; 2016).

**Figure 2.**
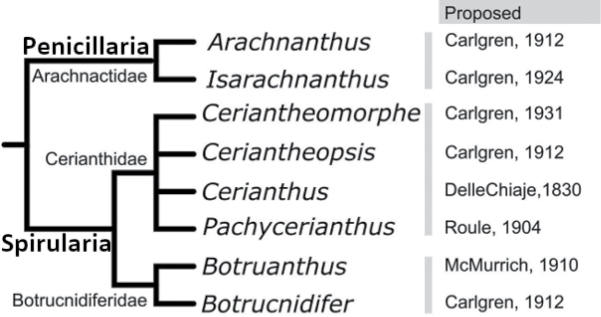
Classification of Ceriantharia (adapted from Stampar et al. 2016a).

Knowledge on Ceriantharia dates back from the late 1700s, with the description by Spallanzani (1784) of an aberrant and tubular hydroid species with a membranous tube around the body, *Tubularie* (= *Tubularia membranosa* in Gmelin, 1791: 3836). Currently, the subclass Ceriantharia is divided into three families, eight genera, and includes 54 valid species (den Hartog 1977; Stampar et al. 2016; Molodtsova 2020) (Fig. 2). Different Ceriantharia classification schemes exist, with only limited consistency with each other (e.g., Mejia et al. 2019). However, until now, there has been only one published table-style catalogue (= nomenclator) of all species of tube-anemones, which is over a century old (Carlgren 1912a) and does not contain an extensive bibliographic compilation. Additionally, many species descriptions (e.g., Arai 1965; Molodtsova 2001a) and higher-level taxonomic reorganizations have taken place since 1912, and, thus, subsequent researchers have faced difficulties in finding and organizing historical and recent citations and information on Ceriantharia. Therefore, there is an obvious need for an organized species nomenclator, as well as for an identification key to aid in further studies of this group. Here, we present a nomenclator and a key to the extant valid Ceriantharia species in their adult form (polyps), including discussions of the status of each species. Some other articles have addressed larval forms (Molodtsova 2004b; Stampar et al. 2015b) but these are not included in the present study.

## Material and methods

The checklist’s classification follows den Hartog (1977) for the orders and Carlgren (1912b, 1931) for genera of the subclass Ceriantharia. Cited articles have mostly been compiled from specific literature. The online databases Hexacorallians of the World (Fautin 2013) and the World Register of Marine Species (Molodtsova 2020) were used to assist in bibliographic compilations. Information on deposited specimens was obtained directly from the original descriptions and/or collections of mentioned museums for each species. If the type material was not found, this information is indicated for each species. In terms of life cycle, larval forms of cerianthids were not added to this study, as it is still not possible to make proper correlations with adult forms without the aid of molecular-based re-examinations (e.g., Stampar et al. 2015c). The identification key was constructed with the characters available for each species, although unfortunately some species have sparse descriptions and only a few distinctive characters are known.

## Results

**Figure 3.**
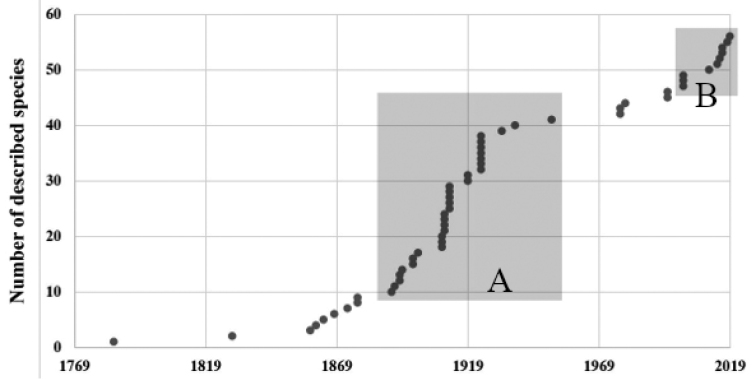
The cumulative number of Ceriantharian species descriptions per year. **A** Period Carlgren/van Beneden/McMurrich (1890 to 1951) and **B** Period Molodtsova/Stampar (2001 to present).

The compilation of species resulted in a list of 54 valid species (Fig. 2) of adult forms of Ceriantharia. The current classification divides the species into the families Arachnactidae (nine species, 16%); Botrucnidiferidae (four species, 8%), and Cerianthidae (41 species, 76%). There is a clear prevalence in numbers of Cerianthidae species, and this can be explained by the large sizes and high visibility of many species described in this family (Stampar et al. 2016). There are two clear periods of comparatively high rates of formal species descriptions with a long break in between them (Fig. 3): a) the ‘Carlgren/van Beneden/McMurrich period’ (1890 to 1951), and b) the ‘Molodtsova/Stampar period’ (ongoing from 2001). During these two periods more than 75% of the valid Ceriantharia species were described.

### Checklist

**Phylum Cnidaria Hatschek, 1888**

**Class Anthozoa Ehrenberg, 1834**

**Subclass Ceriantharia Perrier, 1893**

**Order Spirularia den Hartog, 1977**

Number of valid taxa: two families, six genera, and 45 species

**Family Cerianthidae Milne Edwards & Haime, 1851**

Number of valid taxa: four genera and 41 species

#### Genus Ceriantheomorphe

Taxon classificationAnimaliaSpirulariaCerianthidae

Carlgren, 1931

25D3F550-EE4F-5F2D-BF4F-F02948E42E39

Table 1

##### Type species.

*Ceriantheomorphe brasiliensis* by original designation (Carlgren 1931).

Number of valid species: 3

**Table 1.** Comparison of anatomical features of *Ceriantheomorphe* species (after Lopes et al. 2019).

	*C. ambonensis*	*C. brasiliensis*	*C. adelita*
**Marginal tentacles**	More than 100	Up to 392	Up to 352
**Directive labial tentacle**	Absent	Present	(?)
**Arrangement of labial tentacles**	(0)112.2112	(1)123.1324.3124.3124	(?)112.211
**Actinopharynx**	1/8 to 1/10 of gastric cavity	1/4–1/5 of gastric cavity	1/7 to 1/8 of gastric cavity
**Oral disc**	~ 4 cm	~5 cm	~3 cm
**Siphonoglyph**	Long, 6 mesenteries attached	Rather wide, 4 mesenteries attached	Long, 6 mesenteries attached
**Directive mesenteries**	>Actinopharynx	>Actinopharynx	>Actinopharynx
**P2**	Almost to aboral pole	To aboral pole	Almost to aboral pole
**P3**	Short, ≅ directives	Short, < directives	Short, > directives
**M**	± 2B	± 2B	± 2B
**M3**	± M2	=M2	± M2
**Cnido-glandular tract at fertile mesenteries of first quartets**	Present	Present	Present
**Craspedion tract at fertile mesenteries**	2/4	2/5 – 3/5	2/5 – 3/5

#### 1 Ceriantheomorphe ambonensis

Taxon classificationAnimaliaSpirulariaCerianthidae

(Kwietniewski, 1898)

05455600-C9C5-5C6C-ACD3-AEEF261C24A5

http://zoobank.org/A5A81C1F-3180-4114-A624-E9F1CDA889B5

Cerianthus ambonensis Kwietniewski, 1898: 426; Pax 1910: 167; McMurrich 1910: 26–28 Carlgren 1912a: 44–47; Lopes et al. 2019: 127–148
(?) Cerianthus sulcatus McMurrich, 1910: 28–30
Ceriantheomorphe ambonensis : Carlgren 1931: 1

##### Type locality.

Moluccas (Maluku) Islands, Indonesia, shallow waters.

##### Distribution.

Only known from shallow water at the type locality.

##### Remarks.

The original description made by Kwietniewski (1898) is very simple and based only on external characters. McMurrich (1910) obtained two specimens from the same area and conducted a more detailed anatomical study. Carlgren (1931), based on McMurrich’s description, moved the species to the newly erected genus *Ceriantheomorphe*. There has been no other subsequent research performed on this species. This species is commercially exploited for the international aquarium trade (exported from Indonesia), perhaps owing to its vivid orange color (S. Stampar pers. obs.).

##### Type material.

Not found in this study.

#### 2 Ceriantheomorphe brasiliensis

Taxon classificationAnimaliaSpirulariaCerianthidae

(Mello-Leitão, 1919)

14B85C44-45E3-52E0-AC97-86F51D63B0FF

http://zoobank.org/6336C2A6-B8DB-4B0F-9137-3525DCE698D6

(?) Cerianthus americanus: Hertwig, 1882: 110 116
Cerianthus brasiliensis Mello-Leitão, 1919: 38–39
Ceriantheomorphe brasiliensis : Carlgren 1931: 2–6; Carlgren 1940: 6,11–12; Stampar et al. 2010: 205–209; Silveira and Morandini 2011: 3; Rodriguez et al. 2011: 52, 54–55; Spier et al. 2012: 1–3; Stampar et al. 2012: 5–6, 9; Stampar et al. 2014a: 2,5,8; Stampar et al. 2014b: 344, 347, 351, 353; and Stampar et al. 2015a: 3; González-Muñoz et al. 2016: 5, 9; Stampar et al. 2016: 64, 67, 68; Stampar and Morandini 2017: 690; Lopes et al. 2019: 127–148
(?) Cerianthromorphe brasiliensis: Hedgpeth 1954: 286

##### Type locality.

Baía de Guanabara, Rio de Janeiro, Brazil.

##### Distribution.

Brazil (Espírito Santo (20.5°S) to Rio Grande do Sul (33.7°S) states); Uruguay (35°S), and Gulf of Mexico (dubious record, 24–29°N), shallow waters (at < 40 m depth).

##### Remarks.

This species was first described as *Cerianthus brasiliensis* by Mello-Leitão (1919) from Guanabara Bay, Rio de Janeiro, Brazil. This description is quite simple and based on only a few external morphological characters. Oskar Carlgren visited the Museu Nacional do Rio de Janeiro (MNRJ) at the time of description of the genus *Ceriantheomorphe* sometime between 1925 and 1929, but he was unable to find the type material designated by Mello-Leitão. However, the type material (MNRJ 100) of *Cerianthus brasiliensis* is available and, based on our examinations, every mesentery, except for the directives, is fertile. Thus, based on this, the species described by Mello-Leitão should be moved to the genus *Ceriantheomorphe*. Carlgren (1931) mentions that the species (with this name) as described by him in that publication may be a junior synonym of *C. brasiliensis*Mello-Leitão 1919, as Lopes et al. (2019) have shown. Hertwig (1882) recorded a specimen of *Cerianthus americanus* from the Uruguayan coast whose external shape appeared to be very similar to that of *Ceriantheomorphe brasiliensis*. However, that specimen was not available at the time of the present study and may be lost.

##### Type material.

Museu Nacional do Rio de Janeiro – MNRJ 100 (Holotype).

#### 3 Ceriantheomorphe adelita

Taxon classificationAnimaliaSpirulariaCerianthidae

Lopes, Morandini & Stampar in Lopes et al., 2019

8B7EF140-DDBA-5F33-8DFD-81DCB0177EB0

http://zoobank.org/702bdfdd-870c-43eb-b59a-05a994177d56

Ceriantheomorphe adelita Lopes et al., 2019: 127–148
Cerianthromorphe brasiliensis : Molodtsova 2009: 365–367
(?) Cerianthromorphe brasiliensis: Carlgren and Hedgpeth 1952: 148, 169–170; Hedgpeth 1954: 286; 290; Frey 1970: 309

##### Type locality.

off Port Aransas, 32 km south off Corpus Christi, Texas, United States of America.

##### Distribution.

Gulf of Mexico (Northern Mexico) to North Atlantic (North Carolina, United States of America), shallow waters.

##### Remarks.

A very large species, which for many years was considered to be synonymous with *C. brasiliensis* even without biogeographic justification. Recently, Lopes et al. (2019) have indicated morphological differences that support the distinction between both species. Apparently, this is a species with very low incidence, since several sampling attempts have been unsuccessful (S. Stampar pers. obs.).

##### Type material.

Smithsonian National Museum of Natural History NMNH 50015 (holotype).

#### Genus Ceriantheopsis

Taxon classificationAnimaliaSpirulariaCerianthidae

Carlgren, 1912

F838C430-A60C-56B0-B4AE-F20E4E0B6673

Table 2

##### Type species.

*Ceriantheopsis americana* by original designation (Carlgren 1912a)

Number of valid species: 4

**Table 2.** Comparison of anatomical features of *Ceriantheopsis* species (after Stampar et al. 2016).

	*C. americana*	*C. nikitai*	*C. austroafricana*	*C. lineata*
**Marginal tentacles**	Up to 100–120	Up to 70	Up to 70	Up to 60
**Directive labial tentacle**	Present	Present	Present	Absent
**Arrangement of labial tentacles**	(2)413.4232.4312* (4)413.4231.4312.4312	(3)423.4232.4312.4312	(2)313.4343.4324.3124	4231.4231.4231.4231
**Actinopharynx**	1/12–1/8 of gastric cavity	1/5–1/4 of gastric cavity	1/10–1/8 of gastric cavity	1/6-1/5 of gastric cavity
**Oral disc**	0.7–1.0 cm	~0.6–0.7 cm	Wide, ~1.5 cm in preserved	1.0 – 1.5 cm in preserved
**Siphonoglyph**	Narrow, 4 mesenteries attached	Wide, 4 mesenteries attached	Wide, 4 mesenteries attached	Narrow, 2 mesenteries attached
**Directive mesenteries**	<Actinopharynx	~Actinopharynx	~Actinopharynx	<Actinopharynx
**P2**	To aboral pole	To aboral pole	To aboral pole	Almost to aboral pole
**P3**	=B	=B	=B	=B
**M**	>>B	<2B	>B	≥B
**M3**	≤M2	>M2	≤M2	<Half M2
**Cnido-glandular tract at fertile mesenteries of first quartets**	Present	Not present	Present	Present
**Craspedion tract at fertile mesenteries**	6/7–8/9	3/5	6/7	~6/7-8/9
**Cnido-glandular tract at B**	<<b	=b	<b	<b
**Craspedonemes of craspedion at fertile mesenteries**	Sometimes present	Absent	Absent	Absent

#### 4 Ceriantheopsis americana

Taxon classificationAnimaliaSpirulariaCerianthidae

(Agassiz in Verrill, 1864)

076776A2-F38E-5792-898F-C6A7C95B3002

http://zoobank.org/a5de3b9f-56d7-4f46-a5ff-56a01f2c132a

Cerianthus sp. Agassiz 1859: 24
Cerianthus americanus Agassiz in Verrill, 1864b: 32–33; Verrill 1864a: 56–57; Verrill 1872: 436; Hertwig 1882: 110,116; Andres 1883: 352; McMurrich 1887: 63; van Beneden 1897: 140; Haddon 1898: 401; Parker 1900: 756; Duerden 1902a: 329–330; Duerden 1902b: 301; Roule 1905: 89; McMurrich 1910a: 11,15–16,20; Hargitt 1912: 249; Mello-Leitão 1919: 36–38; van Beneden 1924: 91; Field 1949: 5–6, 18–21, 27; den Hartog 1977: 212; Pei 1998: 179–180
Ceriantheopsis americanus : Carlgren 1912a: 366; Carlgren 1912b: 19–26; Pax 1924: 118; Carlgren 1940: 6, 12–13; Leloup 1964: 257; Frey 1970: 309–311; Peteya 1973a: 301–316; Peteya 1973b: 1–10; Widersten 1976: 858; Shepard et al. 1986: 625–646; Kristensen et al. 1991: 590–591, 589–614; Sebens 1998: 13, 16, 21, 57; Holohan et al. 1998: 466–468; Molodtsova 2000: 14,17; Molodtsova et al. 2011: 2–3, 5–7; Reft and Daly 2012: 123–125, 127, 129; Mata et al. 2012: 602–603; Kayal et al. 2013: 3, 5–6, 10–11, 15; Stampar et al. 2014a: 2, 8
Cerianthiopsis americanus : Hedgpeth 1954: 286–290
Ceriantheopsis americana : Stampar et al. 2015b: 1–6; Stampar et al. 2016a: 69

##### Type locality.

Off Charleston, South Carolina; Beaufort, North Carolina, United States of America (not specified).

##### Distribution.

Atlantic coast of United States and Canada, Gulf of Mexico, and Caribbean Sea, at 2–250 m depth.

##### Remarks.

This species is probably the most extensively studied among the Ceriantharia. There are appropriate descriptions of specimens (McMurrich 1910; Carlgren 1912a) and there is much biological information, especially related to ecological aspects (Shepard et al. 1986; Kristensen et al. 1991; Holohan et al. 1998). One important issue that still needs be studied is the possible occurrence of this species in the deep sea. Several photographic records, especially from ROV surveys below 400 m depth are available on the internet, but, to date, no such deep specimens have been collected for study.

##### Type material.

Museum of Comparative Zoology (Harvard) – Invertebrate Zoology 243 and SCOR-1245 and Peabody Museum of Natural History (Yale) – YPM IZ 000977.CN (syntype).

#### 5 Ceriantheopsis austroafricanus

Taxon classificationAnimaliaSpirulariaCerianthidae

Molodtsova, Griffiths & Acuña, 2011

977DF180-E643-5868-B01D-7ADB77B336FB

http://zoobank.org/5E824C53-474E-4822-B48E-EA47D6801DC0

Ceriantheopsis austroafricanus Molodtsova et al., 2011: 1–7; Stampar et al. 2015b: 1–3, 6

##### Type locality.

Off Cape Town, South Africa.

##### Distribution.

Only known from shallow waters at the type locality (8–15 m depth).

##### Remarks.

This species was recently described and therefore little is known about it beyond a detailed morphological description. One of the most interesting features of the species is the wide range of colors (Molodtsova et al. 2011). This species occurs in waters around Cape Town. Interestingly, this species is found close to industrialized coastal development, such as marinas and ports (S. Stampar pers. obs.), which may have provided a special habitat.

##### Type material.

Zoological Museum of Moscow State University – ZMMU No. Ec-105 (holotype).

#### 6 Ceriantheopsis lineata

Taxon classificationAnimaliaSpirulariaCerianthidae

Stampar, Scarabino, Pastorino & Morandini, 2015

4963EB2C-2690-5C11-8FAC-92D39DCB130D

http://zoobank.org/DD01594B-5E3E-4F92-9877-96AF05CCDE2C

Ceriantheopsis lineata Stampar et al., 2015c: 1475–1481

##### Type locality.

off Quequén, Buenos Aires, Argentina.

##### Distribution.

Warm temperate south-western Atlantic, from Argentina (Buenos Aires State) to Brazil, Laje de Santos (São Paulo State), at 5–130 m depth.

##### Remarks.

This species was recently described, and little is known beyond a detailed morphological description. Similar to *Ceriantheopsis austroafricanus*, this species shows considerable variation in color pattern (Stampar et al. 2015b). The deepest record of the species is 130 m from a dredging expedition (Stampar et al. 2015b). However, it is possible that the species occurs at even greater depths.

##### Type material.

Museu de Zoologia da Universidade de São Paulo – MZUSP 2686 (Holotype).

#### 7 Ceriantheopsis nikitai

Taxon classificationAnimaliaSpirulariaCerianthidae

Molodtsova, 2001

B0EDF20A-6A21-5E58-BAE5-DC5914EE532D

http://zoobank.org/References/F11F4732-5C5A-4A8D-BDDE-13D8888B11DC

Ceriantheopsis nikitai Molodtsova, 2001a: 773–780; Stampar et al. 2015c: 1475, 1480

##### Type locality.

Benguela Upwelling System, Namibia.

##### Distribution.

Only known from deep water at the type locality (145–240 m depth).

##### Remarks.

This species was recently described and has not been the subject of any study since the original description of the species by Molodtsova (2001a). Recorded only from a restricted area in Namibia, this species occurs sympatrically with two other species, *Botrucnidifer shtokmani* and *Cerianthus malakhovi*. These three species were found in the same upwelling system, which suggests that they can also occur in deeper areas.

##### Type material.

Zoological Museum of Moscow University – ZMMU UE-97 (Holotype).

#### Genus Cerianthus

Taxon classificationAnimaliaSpirulariaCerianthidae

Delle Chiaje, 1841

BB15A0D9-D27D-5FA6-978A-7DC1DA4E9B21

Table 3

##### Type species.

*Cerianthus membranaceus* (Spallanzani, 1784)

Number of valid species: 18

**Table 3.** Comparison of anatomical features of *Cerianthus* species.

Species	Directive mesenteries length	Directive labial tentacle	M-mesentery (M1) length	M-mesentery (M2) length	M-mesentery (m1) length	M-mesentery (m2) length	Mesenteries attached to siphonoglyph	Siphonoglyph shape	Number of marginal tentacles
***C. andamanensis***	–	–	Reach aboral pore	–	–	–	–	–	~160
***C. bathymetricus***	–	–	Reach aboral pore	1/2 of M-1	–	–	–	Wide?	28
***C. filiformis***	> stomodeum	Present	Reach aboral pore	6/8 of M-1	7/8 of M-1	5/8 of M-1	6	Wide?	~70
***C. incertus***	–	–	–	–	–	–	–	–	38-42
***C. japonicus***	> stomodeum	Present	Almost reach aboral pore	≅ M-1	3/4 of M-1	~1/2 of M-1	4?	Wide	65
***C. lloydii***	> stomodeum	Present	Almost reach aboral pore	Longer than M-1	1/5 of M-1	1/6 of M-1	4	Narrow	Up to 70
***C. malakhovi***	?		Half column (?)	> M-1	?	?	?	?	~160
***C. medusula***	?	?	?	?	?	?	?	?	Few
***C. membranaceus***	> stomodeum	Present	Almost reach aboral pore	≅M-1	≅P2	≅m-1	6	Narrow	140
***C. mortenseni***	>stomodeum	Present	Short, almost half of gastrovascular cavity	≅ M-1	3/4 of M-1	1/2 of M-1	8	Wide	125
***C. punctatus***	> stomodeum	Present	Almost reach aboral pore	=M-1	2/3 of M-1	1/4 of M-1	6	Rather wide	80-90
***C. roulei***	?	?	Long?	?	?	?	?	?	~40
***C. stimpsonii***	?	?	?	?	?	?	?	?	?
***C. sulcatus***	> stomodeum	Present	Reach aboral pore	≅M-1	?	?	?	Narrow	~180
***C. taedus***	> stomodeum	Present	Short?	Short	?	?	?	Narrow	55
***C. valdiviae***	≅stomodeum	Absent	Short?	=M-1	≅M-1	1/2 of M-1	4	Narrow	35
***C. vas***	?	?	?	?	?	?	?	?	?
***C. vogti***	> stomodeum	Present	(?)Almost reach aboral pore	(?)Longer than M-1	(?)1/5 of M-1	(?)1/6 of M-1	?	Narrow	30-40

#### 8 Cerianthus andamanensis

Taxon classificationAnimaliaSpirulariaCerianthidae

Alcock, 1893

E7EFA931-A2A3-5476-9746-EB8982681C7E

http://zoobank.org/0767B24E-372F-4A41-B206-BE26CF12FE37

Cerianthus andamanensis Alcock, 1893: 153; Carlgren 1896: 174; Pax 1910: 167; Molodtsova 2001b: 913
(?) Cerianthus andamanensis: Haldar 1981: 60–61

##### Type locality.

off Port Blair, Andaman and Nicobar Islands, India.

##### Distribution.

Only known from shallow water at the type locality.

##### Remarks.

The species description is based on three specimens from Port Blair in the Andaman Sea (Alcock 1893); it is very simple with scant information on anatomy. The only two useful pieces of published information are related to the size of the preserved specimens (up to 10 cm in length), and the number of marginal tentacles (up to 160). The specimens observed by Alcock (1893) should be placed within the family Cerianthidae, but currently it is not possible to state that this species is truly part of the genus *Cerianthus*, and a systematic examination of this species is needed. The material recorded by Haldar (1981) is probably not the same species since the biogeographical region is different, but owing to the absence of a detailed description no definite conclusions can be drawn.

##### Type material.

(?) Indian Museum.

#### 9 Cerianthus bathymetricus

Taxon classificationAnimaliaSpirulariaCerianthidae

Moseley, 1877

EB3B28B4-4799-534D-9485-8F5CAB9FD3EA

http://zoobank.org/6078abf1-2d50-4e1f-b956-2cee43bb5f87

Cerianthus bathymetricus Moseley, 1877: 302–305; Andres 1883: 350; van Beneden 1897: 142; Pax 1910: 167; Mello-Leitão 1919: 37; Molodtsova 2000: 14–15, 17, 19; Molodtsova 2001b: 913

##### Type locality.

Deep sea, North Atlantic (35° 26’N 50° 53’W), at 5000 m depth.

##### Distribution.

Only known from deep water at the type locality.

##### Remarks.

This species is one of the smallest tube-dwelling anemone species known. The described specimens are only 2.5 cm long and lived in a very long membranous tube of more than 11 cm in length. The description is not detailed but provides some information on the anatomy, indicating a very long hyposulcus (especially in figure 17, Moseley 1877). Molodtsova (2001b) proposed that this species should be placed within the family Arachnactidae, however we are uncertain about this classification as some species of Cerianthidae have comparatively long hyposulcus regions, and the small size of this species may be misleading. Additionally, the description of the tube is much more consistent with the organization of Cerianthidae (see Stampar et al. 2015a). Thus, we choose to maintain this species as valid until additional studies say otherwise.

##### Type material.

Not found in this study, but the original description provided a graphic representation.

#### 10 Cerianthus filiformis

Taxon classificationAnimaliaSpirulariaCerianthidae

Carlgren, 1924

BA515290-0FB4-5201-9F7C-0D360FDE947C

http://zoobank.org/E466344E-55C1-4CE8-B84F-27BC677961F9

In part Cerianthus orientalisVerrill 1865: 151
Cerianthus sp. 1 Wassilieff 1908: 46
Cerianthus sp. 2 Wassilieff 1908: 46
Cerianthus filiformis Carlgren, 1924: 169–173; Uchida 1979: 195–197; Song 1986: 79–87; Song and Lee 1998: 239–240; Song 1998: 195–198; Pei 1998: 179–180; Song 2000: 324–326; Uchida and Soyama 2001: 127, 150, 152
Cerianthus misakiensis Nakamoto, 1923: 167

##### Type locality.

Aburatsubo Bay, Miura, Japan.

##### Distribution.

South Japan, South Korea, Korea (East China Sea), and China (Yellow Sea), at 1–50 m depth.

##### Remarks.

There are some detailed descriptions about this species (e.g., Nakamoto 1923; Carlgren 1924). Uchida (1979) described the color variation of specimens from Japan (Kushimoto) and some ecological aspects. The same author also compared *C. filiformis* to other specimens described from the same area and concluded that all specimens belonged to the same species. This assumption includes the specimen from Okinose Bank, Sagami Bay (Kanagawa, Japan) described by Wassilieff as *C. orientalis* Verril, 1865. This specimen is deposited in the Zoologische Staatssammlung München (ZSM; 173/D65) and does not belong to the genus *Cerianthus* but to *Ceriantheomorphe* (S. Stampar pers. obs.). It is not very well preserved, but the overall organization of the mesenteries is quite consistent with that of *Ceriantheomorphe ambonensis* (Kwietniewski, 1898). The occurrence of *Ceriantheomorphe* in Japanese waters was also discussed by Molodtsova (2001b).

##### Type material.

Lund Museum of Zoology – MZLU L930/3095b (Syntype).

#### 11 Cerianthus incertus

Taxon classificationAnimaliaSpirulariaCerianthidae

Carlgren, 1932

5AAA9F49-504C-50C9-A124-5A8F4A88B133

http://zoobank.org/8570871C-238B-47B6-A113-4FD870CEF0C5

Cerianthus danielsseni Levinsen, 1893: 398; Carlgren 1896: 174; Roule, 1904: 792; Kingsley 1904: 347; Roule 1905: 85–89; Pax 1910: 167; Carlgren 1912a: 5; Carlgren 1942: 71
Cerianthus incertus Carlgren, 1932: 255; Molodtsova 2000: 14–15; Molodtsova 2001b: 913; Molodtsova 2014: 100

##### Type locality.

North Sea (not specified).

##### Distribution.

Arctic Ocean, Norway, and Iceland, at 650–1185 m depth.

##### Remarks.

*Cerianthus incertus* has a complicated taxonomic history and was originally described as *C. danielsseni* by Levinsen (1893) based only on its external morphology. Carlgren (1896) discussed this problem and, later, Roule (1905), based on specimens from nearby locations, described a new species using the same name. However, at this point, the name *C. danielsseni* became a homonym and was no longer available according to the rules of the International Commission on Zoological Nomenclature (ICZN). Thus, Carlgren (1932) suggested a new name (*Cerianthus incertus*) to solve both situations. Molodtsova (2014) postulated that *C. incertus* is a junior synonym of *C. vogti*, but there are no data available to confirm this hypothesis. Despite discussions on the past taxonomic confusion, this species is still understudied.

##### Type material.

Not found in this study.

#### 12 Cerianthus japonicus

Taxon classificationAnimaliaSpirulariaCerianthidae

Carlgren, 1924

0180D913-E7BD-555E-AD14-D242E1D8BE82

http://zoobank.org/116CDC46-02C7-4B61-B791-F1228F8AD3D3

Cerianthus japonicus Carlgren, 1924: 173–175, Uchida 1979: 185–194; Molodtsova 2000: 19; Molodtsova 2001b: 913

##### Type locality.

Aburatsubo, Misaki (Sagami Bay), Japan.

##### Distribution.

Sagami Bay and Miyazaki, Kyushu Island, Japan; North Hamgyong Province, North Korea, at 10–100 m depth.

##### Remarks.

The original species description was based on two small specimens, one from North Korea (North Hamgyong Province) and the other one from Japan, Aburatsubo, Misaki (Sagami Bay). The description is quite adequate and presents the most important characteristics of the species. However, as noted by Molodtsova (2001b), differences between *C. japonicus* and *C. punctatus* Uchida, 1979 are very subtle. There are only two reliable characters that can be used to differentiate the two species: (1) the organization of the tentacular pseudocycles and (2) the number of mesenteries attached to the siphonoglyph. The first is a plastic character and intraspecific variation has been reported (Carlgren 1912a; Arai 1965), while the second seems to be consistent (Stampar et al. 2016). However, figure 4 by Carlgren (1924) does not allow verification if the third pair of mesenteries (P3) is in contact with the siphonoglyph or not. If the siphonoglyph format is the same as indicated by Uchida (1979), the P3 is probably also connected. Most data indicate that *C. punctatus* is synonymous to *C. japonicus*, which currently cannot be confirmed based on the available data.

##### Type material.

Museum of Evolution- Evolutionsmuseet (Uppsala University – ZTY 2516) (Holotype).

#### 13 Cerianthus lloydii

Taxon classificationAnimaliaSpirulariaCerianthidae

Gosse, 1859

F0D908F8-D16C-54B6-8EAB-393ACB09A400

http://zoobank.org/F0DC99E3-1FB9-4729-9316-01535953DD3A

Edwardsia vestita Gosse, 1856a: 74–75
Cerianthus membranaceus Gosse, 1858: 419
Cerianthus lloydii Gosse, 1859: 50; Gosse 1860: 268–274; Fischer 1874: 201; M’Intosh 1875: 38–39; Robertson 1876: 25, 30; Koren and Danielssen 1877: 80; Andres 1883: 554; Hartlaub 1884: 203; Fischer 1887: 383–384, 432, 437; Carlgren 1893: 120–123, 133, 148; van Beneden 1897: 139–140, 142; Fowler 1897: 806; Haddon 1898: 401; Gravier 1902: 592; Gravier 1904: 259, 267, 276, 278, 280, 288; Roule 1904: 791–792; Roule 1905: 83–85; Walton 1908: 215, 225–226; Torrey and Kleeberger 1909: 117; Pax 1910: 166; McMurrich 1910: 10–11, 17–18; Carlgren 1912a: 11–18; Mello-Leitão 1919: 36, 39; Gravier 1922: 88–89; van Beneden 1924: 101–116, 126, 154; Pax 1928: 201, 234; Stephenson 1928: 83–84; Carlgren 1928: 255–263; Carlgren 1931: 10; Leloup 1931: 2, 3, 5–9; Carlgren 1932: 264–266; Müllegger 1938: 2, 12, 13; Carlgren 1940: 9, 10; Carlgren 1942: 69–71; Nyholm 1943: 95–140, 193–227; Dons 1945: 20; Carlgren 1945: 67–69, 71, 155; Teissier 1950: 33; Williams 1954: 52; Füller 1957: 31; Robins 1969: 339; Riemann-Zürneck 1969: 170, 199, 201, 210, 211, 225; Cutress 1961: 80; Laverack and Blackler 1974: 28; Manuel 1977: 484; den Hartog 1977: 233; Uchida 1979: 194; Manuel 1981: 64–66; Ates 1982: 80–83; Braber and Borghouts 1977: 16, 17; Eleftheriou and Basford 1983: 147–157; Ates 1985: 230–232; Chintiroglou and Koukouras 1991: 395; Harms 1993: 16; Molodtsova and Malakhov 1995a: 5–16; Molodtsova and Malakhov 1995b: 4–11; Ates 1997: 10, 18, 24, 25; Sebens 1998: 48; Blanco 1987: 198; Vafidis and Koukouras 1998: 123; Moore and Cameron 1999: 369–370; Molodtsova 2000: 3, 7, 12–13, 17; Molodtsova 2001a: 778; Molodtsova 2001b: 919; Molodtsova 2001c: 1035; Grebel’nyi 2001: 36; Uchida and Soyama 2001: 129, 150, 152; Molodtsova 2001d: 9, 10; Molodtsova 2003: 252; Molodtsova 2004a: 297; Molodtsova 2004b: 261; Wieking and Kröncke 2005: 395–396; Brown and Collier 2007: 207; Rehm and Rachor 2007: 130, 132; Schückel et al. 2010: 5–7, 9; Bolam et al. 2011: 2239, 2241; Strain et al. 2012: 63–65; Sciberras et al. 2013: 91, 93, 95; Peckett et al. 2014: 336; Coolen et al. 2015: 87
(?) Cerianthus borealis Danielssen, 1860: 251; Verrill 1873b: 405, 414; Danielssen 1888: 1–12; van Beneden 1924: 91, 120–127, 128–131
Cerianthus vermicularis Lütken, 1860: 199–200
Cerianthus lutkenii Andres, 1883: 353
Arachnactis bournei Fowler, 1897: 805–807 (larval stage)
Cereanthus lloydii Goette, 1897: 293
Cerianthus lloydii borealis Grieg, 1913: 142
Synarachnactis bournei Leloup, 1962: 2–4, 6–7 (larval stage)
Cerianthus septentrionalis van Beneden, 1924: 120: 126–131; Molodtsova 2001d: 9–10
(?) Cerianthus sp. Tarasov et al. 1990: 1–3; 6; 15, 17
(?) Cerianthus lloydii: Kussakin and Kostina 1996: 207; Çinar et al. 2014: 684

##### Type locality.

Menai Strait, Irish Sea, United Kingdom.

##### Distribution.

North Sea, Norwegian Sea, Barents Sea, Greenland Sea, Bay of Biscay, and (?) Sea of Okhotsk; (?) depths from 2 m to the deep sea,

##### Remarks.

This species has been the subject of many studies. There have been several morphological descriptions (e.g., Gosse 1860; Carlgren 1912a; Molodtsova and Malakhov 1995a) and several aspects of its ecology and life cycle have been investigated (e.g., van Beneden 1924; Nyholm 1943; Molodtsova and Malakhov 1995b). However, two points still need further attention. The first is related to the distribution of the species, as the presence of some disjointed records in the Pacific Ocean raise the possibility of a disjunct distribution (e.g., more than one species contained in these records). However, the presence of larvae in plankton for long periods of time (Nyholm 1943) may explain the very large occurrence areas as already known for other Ceriantharia species (Stampar et al. 2015c). Braber and Borghouts (1977) reported the occurrence of this species from an estuary system (salinity around 16 psu), and Çinar et al. (2014) from the coast of Turkey, although the second record is questionable. The second point is related to the position of the species within the genus *Cerianthus*. Preliminary studies (unpublished) based on molecular data indicate that perhaps this species is more related to the genus *Ceriantheopsis* than to *Cerianthus*.

##### Type material.

Not found in this study.

#### 14 Cerianthus malakhovi

Taxon classificationAnimaliaSpirulariaCerianthidae

Molodtsova, 2001

393CBAB5-40C1-5BBF-8BEF-DCBB90509A12

http://zoobank.org/830A9DD9-8EE9-4849-94EA-D89979D06926

Cerianthus malakhovi Molodtsova, 2001a: 909–913; Molodtsova 2001b: 913; Molodstova et al. 2011: 1

##### Type locality.

Close to Torra Bay and Mowe Bay, Skeleton Coast Park, Namibia; at 300–350 m depth.

##### Distribution.

Only known from deep water at the type locality.

##### Remarks.

This species has been described in detail relatively recently based on five collected specimens. The original description, in Russian, contains no information on living animals because the material examined was already fixed at the time of diagnosis. This is a species that requires attention, as it can occur in deeper waters and may contain very important evolutionary information.

##### Type material.

Zoological Museum of Moscow University, ZMMU EC-102 (Holotype).

#### 15 Cerianthus medusula

Taxon classificationAnimaliaSpirulariaCerianthidae

(Klunzinger, 1877)

BB2BDFAB-C529-5ACB-A5CE-DB31E493301E

http://zoobank.org/0D8AA956-7C46-4DCA-889E-32BCFC84F419

Paractis medusula Klunzinger, 1877: 71–72
Cerianthus medusula Andres, 1883: 353–354; Cerfontaine 1891a: 37–38; van Beneden 1897: 141; Mello-Leitão 1919: 36
(?) Pachycerianthus maua: Krempf 1905: 195
(?) Pachycerianthus mana: Fishelson 1970: 109

##### Type locality.

Al-Qusair (Red Sea), Egypt.

##### Distribution.

Only known from shallow water (at < 5 m depth) at the type locality.

##### Remarks.

This is another species with only little available data, and these are quite contradictory. This species was described as a sea anemone (order Actiniaria) by Klunzinger (1877) based only on the external morphology. Andres (1883) described some aspects of the external morphology based on work by Klunzinger (1877) and indicated that this must be a species of family Cerianthidae. Cerfontaine (1891a) argued that this species may be the same as *C. oligopodus* (= *Arachanthus oligopodus*) found in Italy, and furthermore the specimen observed by Klunzinger (1877) was not in good condition. However, it is not possible to make more statements about this species due to the absence of material available from the region. On the other hand, the indication that this species is a member of the family Arachnactidae as stated by Cerfontaine (1891a) seems to be incorrect. The few characters present in the descriptions are not consistent with those of the Arachnactidae, but instead with those of the Cerianthidae. This is a species that requires additional sampling from the type locality for further examination, especially as some specimens identified as *Pachycerianthus maua* Carlgren, 1900 have been subsequently collected from the same region (Krempf 1905; Fishelson 1970).

##### Type material.

Not found in this study, but the original description provided a graphic representation.

#### 16 Cerianthus membranaceus

Taxon classificationAnimaliaSpirulariaCerianthidae

(Gmelin, 1791)

2486D533-3699-57ED-A516-8FD06BDF1D84

http://zoobank.org/3AD7FFEB-56C0-49A3-9BDB-00A813327D90

Tubularie Spallanzani, 1784: 627–628
(?) Tubularie: Rapp 1829: 656–658
Tubularia membranosa Gmelin, 1791: 3836
Actinia cylindrica Renier, 1807: 23
Actinia vestita Renier, 1807: 23–24
Moschata rhododactyla Renier in de Blainville, 1830: 284; de Blainville 1834: 318;
Cereus cupreus Ilmoni, 1830: 698–699; Ilmoni 1831: 123
In part Actinia elongata Grube, 1840: 11–12; Sars 1857: 33
Cerianthus cornucopia Delle Chiaje, 1841: 136; Milne Edwards and Haime 1851: 14
Cerianthus breae Delle Chiaje, 1841: 136
Cerianthus actiniodeus Delle Chiaje, 1841: 136
Cerianthus membranaceus : Haime 1854: 352–389; Milne-Edwards 1857: 309; Sars 1857: 28–32; Agassiz 1863: 529; Fischer 1874: 200–203, 237–239; Fischer 1875: 184–185; Heider 1879: 204–254; Jourdan 1880: 16, 44–45, 103–117, 130–132, 152–153; Andres 1881: 331–332; Andres 1883: 347–349; Mark 1884: 42; Graeffe 1884: 340; Fischer 1887: 383–385, 405, 432, 435; Hertwig 1888: 54; Fischer 1889 252, 254–265; Hickson 1889: 8; Cerfontaine 1891a: 37; Cerfontaine 1891b: 133–141; Goette 1897: 292–316; van Beneden 1897: 54–56, 139, 142; Gravier 1902: 592; Child 1903a: 239–260; Child 1903b: 10; Child 1904a: 70–71; Child 1904b: 279–284; Gravier 1904: 259, 269, 276, 278, 280; Carlgren 1906: 77–78; van Beneden 1924: 18, 28, 59, 30–68, 70, 73, 77, 85, 92, 94, 104, 108, 135–144, 163–164, 175; Carlgren 1927: 443; Menon 1927: 31–32; Torelli 1932: 2–15; Müllegger 1938: 2, 12; Pax and Müller 1955:110; Leloup 1960:1–3; Pax and Müller 1962: 107–110; Leloup 1962: 3–4, 6–7; Schmidt 1972: 427, 429, 431, 433; Schmidt 1974: 535, 537, 547; den Hartog 1977: 233, 235; Uchida 1979: 194; Ates 1982: 80–83; Tur and Godall 1982: 178, 180–182; Gili 1982: 120, 125–126; Ates 1985: 230; Morri et al. 1991: 37; Chintiroglou et al. 1995: 362; Vafidis and Koukouras 1998: 119–120, 123; Molodtsova 2000: 14, 17; Ocaña et al. 2000a: 57; Molodtsova 2001b: 913; Molodtsova 2004: 261; Wiedenmann et al. 2004: 270, 272–274, 276; Calado 2006: 391; Nienhaus et al. 2006: 12942–12943; Casellato et al. 2007: 127, 129; Cosentino et al. 2011: 409–411, 413–414; Lo Iacono et al. 2012: 466–467; Rastorgueff et al. 2015: 142, 148; Stampar et al. 2015b: 2167
(?) Edwardsia vestita Gosse, 1856a: 74–75; Gosse 1856b: 220; Milne-Edwards 1857: 286; Gray 1867: 240
(?) Cerianthus membranaceus: Gosse 1858: 419; Heller 1868: 20, 79
Cerianthus cylindricus Milne-Edwards, 1857: 309; Heller 1868: 20, 79
Saccanthus purpurescens Milne-Edwards, 1857: 310
Cerianthis membranaceus : Sars 1857: 28–32
(?) Cerianthus lloydii Gosse, 1859: 419; Çinar et al. 2014: 684
Cerianthus membranaceus nigricans Andres, 1881: 332
Cerianthus membranaceus violaceus Andres, 1881: 332
Cerianthus membranaceus viridis Andres, 1881: 332
Cerianthus membranaceus roseus Andres, 1881: 332
Cerianthus nans Andres, 1881: 333
Saccanthus purpurascens Andres, 1883: 351
Saccanthus purpurensis van Beneden, 1897: 142
Pachycerianthus multiplicatus : Çinar et al. 2014: 684

##### Type locality.

Mediterranean Sea, Italy (not specified).

##### Distribution.

Mediterranean Sea (Italian coast), shallow waters.

##### Remarks.

This is one of the most well-known cerianthid species, but at the same time there are many questions about the taxonomic consistency of past works. This was the first species of Ceriantharia described (type locality Italy); however, this has caused many records from the Mediterranean Sea being incorrectly attributed to this species. For example, Pax (1908) argued that C. (Saccanthus) maderensis (= *Isarachnanthus maderensis*) Johnson, 1861 is synonym of *C. membranaceus* based on the two species’ original descriptions. However, the descriptions presented and discussed by Johnson (1861) and Pax (1908) are incompatible with *C. membranaceus* and more likely describe a species of the family Arachnactidae, perhaps a member of the genus *Isarachnanthus*. In addition, *I. maderensis* is very common in Madeira and the Azores (Stampar et al. 2012; Stampar and Morandini 2017). Haque (1977) recorded *C. membranaceus* from Bangladesh and Pakistan, but these records are biogeographically incongruent and probably these specimens from the Indian Ocean are another species. In short, *C. membranaceus* is a species that requires a comprehensive review of all its records, especially for the presence of cryptic species, and to assess if the different morphotypes reported truly belong to just one species or to a species complex.

##### Type material.

Not found in this study.

#### 17 (?) Cerianthus mortenseni

Taxon classificationAnimaliaSpirulariaCerianthidae

Carlgren, 1924

47198379-4F64-54DB-AF4E-23CCD2B2BFB0

http://zoobank.org/3AA339E2-4BFC-424A-997A-BBEA7EB45041

Cerianthus mortenseni Carlgren, 1924: 175–182, 195; Molodtsova 2001b: 773

##### Type locality.

Paniquian Island, Mindoro, Philippines.

##### Distribution.

Only known from shallow water at the type locality.

##### Remarks.

This is a very intriguing species as the two specimens described in the original description are very different in shape. The organization of the mesenteries is not coincident on both sides of the body, indicating a considerable difference in the development of mesenteries (Carlgren 1924). This sort of large variation is not commonly reported in tube-dwelling anemones. The mesentery organization and associated structures indicate a strong correlation of these specimens with the genera *Cerianthus* and *Pachycerianthus*. Thus, this species may be important in the ongoing discussion about the validity of the genus *Pachycerianthus* (Torelli, 1961). Unfortunately, these are the only two specimens collected from the type locality (Philippines). Other specimens examined from this area (S. Stampar pers. obs.) are not similar to the specimens described by Carlgren (1924).

##### Type material.

Department of Zoology, University of Stockholm, Sweden (holotype) (?).

#### 18 Cerianthus punctatus

Taxon classificationAnimaliaSpirulariaCerianthidae

Uchida, 1979

EDB26C91-64EE-5A91-8837-A8DF51BB424E

http://zoobank.org/DBEE9A26-932A-4F64-B0D5-12BF79BA5E33

Cerianthus punctatus Uchida, 1979: 189–195; Molodtsova 2000: 19; Uchida and Soyama 2001: 129, 150, 152; Molodtsova 2001b: 913

##### Type locality.

Suruga Bay (Numazu), Japan.

##### Distribution.

Only known from shallow water at the type locality.

##### Remarks.

The available information on this species is amongst the most complete from before the advent of detailed descriptions in the 2000s. Uchida (1979) gives a complete comparison of several characters with most species of the genus *Cerianthus*. However, besides morphological data there is still no other information about this species (e.g., reproduction).

##### Type material.

Saibura Marine Park Research Station (lost?), but the original description provided a graphic representation.

#### 19 Cerianthus roulei

Taxon classificationAnimaliaSpirulariaCerianthidae

Carlgren, 1912

868DA38A-121F-5472-B633-9CF213A0930B

http://zoobank.org/8180341D-C667-45AF-9A80-B85DB5C6B2CD

Cerianthus lloydii Gosse, 1859: 50; Roule 1904: 791–792; Roule 1905: 83–85; van Beneden 1924: 111–116
Cerianthus roulei Carlgren, 1912a: 3–5; Carlgren 1932: 255–256; Carlgren 1942: 71; Molodtsova 2001b: 913

##### Type locality.

close to Svalbard, Norway, Greenland Sea.

##### Distribution.

Svalbard, Norway, Greenland Sea; depth unknown.

##### Remarks.

This species has a very deficient description and is represented by very few museum specimens for comparison. The description of *C. lloydii* by Roule (1905) (see synonymy list) may fit a range of species (e.g., *Cerianthus lloydii*, *Ceriantheopsis americana*) and thus it is not possible to discuss it based on these data. The type locality is difficult to reach, and it may be very problematic to obtain additional specimens due to the absence of precise locality data and because material is likely to be from great depths up to 5000 m (Ritzmann et al. 2004). Therefore, the validity of this species remains uncertain.

##### Type material.

Not found in this study.

#### 20 (?) Cerianthus stimpsonii

Taxon classificationAnimaliaSpirulariaCerianthidae

Verrill, 1868

15F09FE8-1C9A-58EB-B0F3-B9CB23D4BA57

http://zoobank.org/555D3481-FEF5-4615-9C3F-DE58BB8B4EE8

Cerianthus stimpsonii Verrill, 1868: 317–318; Verrill 1870: 102; Andres 1883: 351–352; van Beneden 1897: 140; Pax 1910: 167; Mello-Leitão 1919: 36; Molodtsova 2001b: 913

##### Type locality.

Port Lloyd, Bonin Islands (Ogasawara Islands), Japan.

##### Distribution.

Only known from shallow water (18 m depth) at the type locality.

##### Remarks.

Based on the description by Verrill (1868), this species probably belongs to the family Arachnactidae, particularly due to the description of a soft tube (see Stampar et al. 2015b). The few external characters presented are only consistent with those of the family Arachnactidae. Unfortunately, there are no specimens from the Ogasawara Islands deposited in museums, and further discussions on this point depends on finding some material that can be correlated with the type material or else new collections for the designation of a neotype.

##### Type material.

Not found in this study.

#### 21 Cerianthus sulcatus

Taxon classificationAnimaliaSpirulariaCerianthidae

Kwietniewski, 1898

7A7F422C-FB32-52AB-B29E-3A2FABB9BA89

http://zoobank.org/98D51C07-651B-4476-8409-BE60BA39C86A

Cerianthus sulcatus Kwietniewski, 1898: 427; Pax 1910: 167; Carlgren 1912a: 44–47; Uchida 1979: 194; Molodtsova 2001b: 919
(?) Cerianthus sulcatus: McMurrich 1910: 28–30

##### Type locality.

Raha, Ambon, Moluccas, Indonesia.

##### Distribution.

Only known from shallow water at the type locality.

##### Remarks.

This species was described by Kwietniewski (1898) based on a 4 cm long and 2.5 cm wide specimen with 90 tentacles in each tentacle series (marginal and labial) and three cycles each. However, these are the only characteristics indicated. McMurrich (1910a) gave a description of a specimen collected from near the type locality. However, the description is incomplete and presents some differences compared to original description. Thus, currently, we cannot confirm if this species is valid or not.

##### Type material.

Not found in this study.

#### 22 Cerianthus taedus

Taxon classificationAnimaliaSpirulariaCerianthidae

McMurrich, 1910

1BE603B6-34E0-5EDA-8130-32EBB8685ADA

http://zoobank.org/DBA7107E-9E29-43D3-8345-822BB1D77067

Cerianthus taedus McMurrich, 1910: 30–31; Carlgren 1912a: 44–47; van Soest 1979: 117; Pei 1998: 181

##### Type locality.

Makassar Strait, Central Sulawesi, Indonesia.

##### Distribution.

Only known from deep water (at 724 m depth) at the type locality.

##### Remarks.

This species was described based on only one damaged specimen, which was 6 cm long, with 55 marginal and labial tentacles arranged in two and four cycles, respectively. The organization of the mesenteries was not described in detail by McMurrich (1910), who simply indicated the alternation of fertile and sterile mesenteries. There are several observations of different morphotypes that are not formally associated with any other name described from this or related areas. As there are no other species described for this region with this morphotype, it is probably a valid species, but it is not possible to certainly state that this species belongs to *Cerianthus*.

##### Type material.

Possibly lost (Zoological Museum of Amsterdam, now Naturalis Biodiversity Center, Leiden).

#### 23 Cerianthus valdiviae

Taxon classificationAnimaliaSpirulariaCerianthidae

Carlgren, 1912

CCBC4582-AFFE-5838-96B5-68AA9B32AAB7

http://zoobank.org/15818B70-049E-4801-AFB0-A1D1E69FCA4A

Cerianthus valdiviae Carlgren, 1912a: 44–47; Carlgren 1923: 245–252; Uchida 1979: 193; Molodtsova 2001b: 929

##### Type locality.

Between Keeling and south Sumatra, Indian Ocean.

##### Distribution.

Only known from deep water (at 5000 m depth) at the type locality.

##### Remarks.

This species was initially described in a table by Carlgren (1912a). However, the same author redescribed this species in 1923 in more detail. This is a species from the deep sea; however, the detailed description allows confirmation that this species belongs to *Cerianthus*. This is another example of a species that needs further study, as it may present very different characters compared to species from shallow waters.

##### Type material.

Not found in this study.

#### 24 Cerianthus vas

Taxon classificationAnimaliaSpirulariaCerianthidae

McMurrich, 1893

39584AFB-5E6E-506A-A786-43D36C61A422

http://zoobank.org/c01102cc-ec9d-474a-968e-82b0bbe5686f

Cerianthus vas McMurrich, 1893: 202–203, 206; Carlgren 1896:174; Haddon 1898: 401; Torrey and Kleeberger 1909: 115 -116; Pax 1910: 167; Arai 1965: 205

##### Type locality.

Cedros Island, Mexico (Pacific coast).

##### Distribution.

Only known from shallow to deep water (at 80 m depth) at the type locality.

##### Remarks.

This is a doubtful species, as the original description is very incomplete, and some characters are incongruent. Torrey and Kleeberger (1909) comment that *Cerianthus vas* is a very problematic species, but they did not discuss the problems in detail. This species may actually be valid, but due to the absence of materials from the same depth and location, it is not possible to further discuss this.

##### Type material.

Not found in this study, but the original description provided a graphic representation.

#### 25 Cerianthus vogti

Taxon classificationAnimaliaSpirulariaCerianthidae

Danielssen, 1890

DC19FE72-8AF0-59F8-9F4D-CD20F809D20F

http://zoobank.org/3B09DBC0-6D5E-4371-AAD7-94F8DAA4DABC

Cerianthus vogti Danielssen, 1890: 137–142; Carlgren 1895: 284; Roule 1905: 89; Pax 1910: 167; Carlgren 1912a: 18–21; Mello-Leitão 1919: 36–38; Carlgren 1932: 255; Carlgren 1942: 69, 71; Jensen 1992: 75–80; Molodtsova 2000: 15,17; Molodtsova 2001b: 919
Cerianthus abyssorum Danielssen, 1890: 143; Carlgren 1895: 284; van Beneden 1897: 140; Roule 1905: 89; Pax 1910: 167; Mello-Leitão 1919: 36

##### Type locality.

Norwegian Sea (not specified).

##### Distribution.

Only known from deep water (at 900–1400 m depth) at the type locality.

##### Remarks.

This species is well known, even though it is a species from deeper areas. The description by Danielssen (1890) is incomplete as it presents few characters related to the organization of the mesenteries. Nevertheless, it is quite detailed in various other aspects. Carlgren (1912a) presents a slightly more complete description with more comprehensive information on the organization of the mesenteries and a comparison with *C. lloydii*. Jensen (1992) presents environmental and biological data about the species, especially on the occurrence of branching and fairly long tubes (tube system).

##### Type material.

Not found in this study.

#### Genus Pachycerianthus

Taxon classificationAnimaliaSpirulariaCerianthidae

Roule, 1904

0ED8DDA7-6FBB-5A7F-91CF-03A047F845B8

Table 4

##### Type species.

*Pachycerianthus multiplicatus* Carlgren, 1912a (proposed by Kelly and Keegan 2000)

Number of valid species: 16

**Table 4.** Comparison of anatomical features of *Pachycerianthus* species (after Stampar et al. 2015).

Species	Directive mesenteries length	Directive labial tentacle	M-mesentery (M1) length	M-mesentery (M2) length	M-mesentery (m1) length	M-mesentery (m2) length	Mesenteries attached to siphonoglyph	Siphonoglyph shape	Number of marginal tentacles
***P. aestuari***	> stomodeum	?	Reach aboral pore	≅ M-1	1/5 of M-1	= m-1	16	Wide	30–34
***P. benedeni***	< stomodeum	?	Reach aboral pore	?	?	?	6?	Wide?	~125
***P. borealis***	> stomodeum	?	Reach aboral pore	= M-1	3/4 of M-1	~1/3 of M-1	8	Wide	139–155
***P. curacaoensis***	> stomodeum	Absent	Reach aboral pore	1/2 of M-1	1/4 of M-1	2/3 of m-1	4	Short and narrow	74–105
***P. delwynae***	> stomodeum	Present	Almost reach aboral pore	Larger than M-1	1/3 of M-1	1/2 of M-1	6	Narrow	89–114
***P. dohrni***	?		Half column (?)	> M-1	?	?	?	?	~160
***P. fimbriatus***	> stomodeum	Present	Reach aboral pore	3/4 of M-1	1/3 of M-1	1/3 of M-1	8	Wide and long	<60
***P. insignis***	< stomodeum	Present	Almost reach aboral pore	≅M-1	≅M-1	≅M-2	8	?	~100
***P. johnsoni***	< stomodeum	?	Reach aboral pore	≅3/4 of M-1	3/4 of M-1	1/2 of M-1	8	Wide	~108
***P. longistriatus***	> stomodeum	Present	Reach aboral pore	=M-1	1/3 of M-1	1/4 of M-1	6	Wide	138–140
***P. magnus***	> stomodeum	Present	Almost reach aboral pore	3/4 of M-1	1/3 of M-1	1/2 of M-1	6	Short and narrow	~120
***P. maua***	< stomodeum	Absent	Reach aboral pore	1/4 of M-1?	1/3 of M-1?	1/3 of M-1?	6	Narrow	~150
***P. monostichus***	> stomodeum	Present	Reach aboral pore	≅M-1	1/2 of M-1	≅m-1	8	Narrow and long	~47
***P. multiplicatus***	> stomodeum	Absent	Reach aboral pore	=M-1	1/3 of M-1	1/3 of M-1	6	Narrow	175
***P. nobilis***	?	?	?	?	?	?	?	?	160–170
***P. schlenzae***	> stomodeum	Present	Reach aboral pore	3/4 of M-1	1/2 of M-1	1/3 of M-1	6	Long and narrow	60–85
***P. solitarius***	> stomodeum	Present	Reach aboral pore	≅ M-1	1/4 of M-1	1/5 of M-1	6	Narrow	~64

#### 26 Pachycerianthus aestuarii

Taxon classificationAnimaliaSpirulariaCerianthidae

(Torrey & Kleeburger, 1909)

A286AD64-37B1-541B-9E5A-F5DF7075B7B1

http://zoobank.org/3DEBCB69-32C3-4CDD-8F03-AFBD67DFDBB8

Cerianthus aestuarii : Child, 1908: 27–53; Torrey and Kleeburger 1909: 115–119, 121, 123; Pax 1910: 167; Pei 1998: 181
Pachycerianthus aestuari : McMurrich, 1910: 11; Arai 1965: 205, 210; Arai 1971: 1680; Carter 1995: 6
Pachycerianthus aestuarii : Carlgren 1912a: 44–47; Stampar et al. 2014b: 345, 350, 352

##### Type locality.

Mission Bay, East Pacific, California, United States of America.

##### Distribution.

East Pacific, California, USA, shallow waters.

##### Remarks.

This species was described by Torrey and Kleeburger (1909) based on specimens obtained from Mission Bay, California. This description is not very detailed but relevant information about its morphology is available. Child (1908) described some information on the movements and regeneration of P. (Cerianthus) aestuarii and morphological adaptations in relation to the environment. Carlgren (1912a) moved the species to genus *Pachycerianthus* based on the original description. Arai (1965) described a new species of the same genus, *P. torreyi*, from a nearby area and claimed that this was not the same species as described by Torrey and Kleeburger (1909). This species was considered a synonym of *P. fimbriatus* by Arai (1971), which occurs in the same area as *P. aestuarii*.

##### Type material.

Not found in this study, but the original description provided a graphic representation.

#### 27 Pachycerianthus borealis

Taxon classificationAnimaliaSpirulariaCerianthidae

(Verrill, 1873)

9628F2A1-C13D-5BBD-AEB7-25EB0F68F9FF

http://zoobank.org/73181FBE-06D8-416C-8C79-C94AF7FB94E7

Cerianthus borealis : Danielssen 1860: 251 (senior homonym); Verrill 1873b: 5,14; Verrill 1873a: 349, 350, 368, 391; Verrill 1873c: 440–441; Verrill 1874: 413; Harger and Smith 1876: 54; Verrill 1879: 15; Andres 1883: 352; Verrill 1885: 534; Danielssen 1888 1–12; McMurrich 1893: 204; van Beneden 1897: 140 -142; Parker 1900: 757; Kingsley 1904: 345–359; Torrey and Kleeburger 1909: 119, 125; McMurrich 1910a: 167; Pax 1910: 167; Carlgren 1912a: 44–47; Mello-Leitão 1919: 36; 37; Verrill 1922: 134–136; van Beneden 1924: 91, 120–127, 128–131; MacGinitie 1955: 61, 75, 84, 85, 97, 122; Widersten 1976: 857, 858; Shepard et al. 1986: 625–646; Sebens 1998: 13, 16, 21, 57; Molodtsova 2001d: 9; Molodtsova 2004b: 261
Cerianthus verrillii McMurrich, 1910: 10–11
Pachycerianthus borealis : Molodtsova 2000: 15, 17; Molodtsova 2001b: 9; Stampar et al. 2014b: 344–345, 350, 352–353

##### Type locality.

Georges Bank, Massachusetts, United States/Nova Scotia, Canada (not specified).

##### Distribution.

Northwestern Atlantic (Arctic Sea to North Carolina, USA), at depths of 10–500 m.

**Remarks.** This species was described by Verrill (1873b) based on external morphology but he did not give many details. Danielssen (1888) gave a very detailed description, including various characters concerning the internal anatomy. A century later, Shepard et al. (1986) presented a study on ecological aspects of tube-dwelling anemones from the Northwest Atlantic and included some information about Pachycerianthus (Cerianthus) borealis. Molodtsova (2001b), in her discussion of the genus *Cerianthus*, showed that *Cerianthus borealis* should be part of the genus *Pachycerianthus*. This species occurs at lower temperatures and apparently resists considerable variations in salinity (S. Stampar pers. obs.).

##### Type material.

Peabody Museum of Natural History (Yale – YPM 9830, 9831, 9832 (Syntype).

#### 28 Pachycerianthus curacaoensis

Taxon classificationAnimaliaSpirulariaCerianthidae

den Hartog, 1977

59AAB386-DD32-5F7B-BBA7-3870FA5B73E2

http://zoobank.org/9599F783-723C-4B15-9949-3C80F5AB8B98

Pachycerianthus curacaoensis den Hartog, 1977: 215–221, 237; Carter 1995: 6; Molodtsova 2000: 15, 17; Stampar et al. 2014b: 344, 345, 350, 353

##### Type locality.

Curaçao, Dutch Caribbean.

##### Distribution.

Caribbean Sea (Curaçao), at 65–75 m depth.

##### Remarks.

This species was described by den Hartog (1977) based on specimens from Curaçao. The description of this species is fairly detailed and includes a wide range of biological and morphological information. This is the only species of this genus in the Caribbean Sea and it shows no morphological similarity to congeners described from the Pacific Ocean. On the other hand, this species shares some characters with *P. schlenzae*, which was described from the South Atlantic (Stampar et al. 2014b). Thus, the evolutionary correlation of these two species is of biogeographical relevance.

##### Type material.

Naturalis Biodiversity Center (former Rijksmuseum van Natuurlijke Historie, Leiden – RMNH.COEL.11359 (holotype).

#### 29 Pachycerianthus delwynae

Taxon classificationAnimaliaSpirulariaCerianthidae

Carter, 1995

09D5576D-7C28-5759-BA50-3DF678201A18

http://zoobank.org/96DDF06E-467C-47ED-9DB1-96E8085BC67A

Pachycerianthus delwynae Carter, 1995: 2–3; Molodtsova 2007: 133; Stampar et al. 2014b: 350, 352

##### Type locality.

off Port Jackson, Sydney harbor, Australia.

##### Distribution.

Sydney harbor, Australia, at 5–15 m depth.

##### Remarks.

This is one of two species of this genus described from Australia by Carter (1995), the other one being *P. longistriathus*. They co-occur in the same bay and therefore doubts about the consistency of the two taxonomic species still exist. The morphological differences between the two species appear consistent, but intraspecific variation is quite significant and thus a more thorough evaluation of the morphological characters and the inclusion of molecular data may change this view.

##### Type material.

Australian Museum; AMG15399 (holotype).

#### 30 Pachycerianthus dohrni

Taxon classificationAnimaliaSpirulariaCerianthidae

(van Beneden, 1924)

F2BDD141-DAA2-504E-B83E-A7E09F12BA4A

http://zoobank.org/3C1075B6-988C-4FB5-8855-FD4CC5DCA9D0

Cerianthus membranaceus viridis Andres, 1881: 332
Cerianthus membranaceus Andres, 1883: 347–349
Cerianthus dohrni : Lo Bianco 1909: 552; Pax 1910: 166; van Beneden 1924: 24, 30, 32, 33, 45, 60, 63, 65–89, 92, 94
In part Cerianthus viridis Torelli, 1932: 1–15
Pachycerianthus dohrni : Carlgren 1940: 15; Arai 1971: 1679; Carter 1995: 6; Vafidis and Koukouras 1998: 122–123; Stampar et al. 2014b: 350, 352

##### Type locality.

Naples, Tyrrhenian Sea, Italy.

##### Distribution.

Tyrrhenian Sea, Italy and Aegean Sea, Greece, shallow waters.

##### Remarks.

This species was initially described from the Italian coast (Naples region) as a variation of *Cerianthus membranaceus* (Andres 1881). However, Lo Bianco (1909) recognized distinct differences from the material identified as *C. membranaceus* and suggested a new name, *Cerianthus dohrni*, but without giving a description. Subsequently, van Beneden (1924) gave a very detailed morphological description of the species with some observations from specimens in aquaria. Some years later, Torelli (1932) described *Cerianthus viridis* based on specimens with a morphology clearly related to that of *Cerianthus dohrni*. Carlgren (1940) relocated *C. dohrni* to the genus *Pachycerianthus*. This is one of the largest species of tube-dwelling anemones in the world with a length of more than 40 cm, which is comparable to the lengths of *Ceriantheomorphe brasiliensis* and *Cerianthus membranaceus*.

##### Type material.

Not designated (several specimens mentioned, which can be considered syntypes).

#### 31 Pachycerianthus fimbriatus

Taxon classificationAnimaliaSpirulariaCerianthidae

McMurrich, 1910

B5B1BBB1-D9FE-56AE-9E61-79F8F675972B

http://zoobank.org/A1F21314-9AFC-42C8-A5DE-1A70BB00D3AE

(?) Cerianthus elongatus Kwietniewski, 1898: 426–427; Pax 1910: 167
Pachycerianthus fimbriatus McMurrich, 1910: 35–38; Carlgren 1912a: 44–47; Arai 1971: 1677–1680; Arai 1972: 311–317; Arai and Walder 1973: 1086–1088, 1090; Arai and Karakashian 1973: 719–720, 723–724; Tiffon and Hugon 1977: 289–290; Uchida 1979: 188; Carter 1995: 6; Fautin 1998: 135; Arai 1985: 47–48; Pirtle et al. 2012: 1896, 1905–1906; Stampar et al. 2014b: 350, 352
Pachycerianthus plicatus Carlgren, 1924: 182–186, 195; den Hartog 1997: 352; Arai 1971: 1677; 1680
(?) Pachycerianthus torreyi Arai, 1965: 205–210; Arai 1971: 1677; 1680

##### Type locality.

Cebu, Philippines.

##### Distribution.

Sulu Sea and Celebes Sea, Philippines, and Indonesia, (?) Pacific Coast of US and Canada; shallow waters.

##### Remarks.

This species forms part of a taxonomic problem. The description of *P. fimbriatus* was based on a study of 15 specimens collected mainly from the Celebes Sea, Philippines, by McMurrich (1910). In the same study, McMurrich argued that *Cerianthus elongatus* was the same as the new species *P. fimbriatus*, and considered Kwietniewski’s (1898) description as incomplete and invalid. McMurrich (1910) also argued that *Cerianthus nobilis* described by Haddon and Shackleton (1893), based on specimens from North Australia, could also be the same species, but specimens were not available for comparison. Later, Arai (1965) described a new species from the Pacific Coast of North America, *P. torreyi*. The same author in 1971 recognized that this species was highly correlated with McMurrich’s *P. fimbriatus* from the Celebes Sea. Thus, Arai (1971) considered *P. torreyi* to be a junior synonym of *P. fimbriatus*. However, the geographical distribution is disjunct by 14,000 km, and it is likely that a more detailed study with the inclusion of molecular data will present different results. In our opinion, *P. torreyi* should be a valid species.

##### Type material.

The provenance data of a specimen in the Natural History Museum at London, NHMUK 1889.11.25.64, is coherent with the locality and dates in the original description, but it is impossible to make an exact connection between the materials.

#### 32 Pachycerianthus insignis

Taxon classificationAnimaliaSpirulariaCerianthidae

Carlgren, 1951

F8F6E5B7-E84A-5500-A1EA-4A2B99269C57

http://zoobank.org/046E54F7-B238-4BD9-8E51-0EFA1FD54917

Pachycerianthus insignis Carlgren, 1951: 435–436; Arai 1965: 205, 210; Arai 1971: 1679; Carter 1995: 6; Stampar et al. 2014b: 350, 352

##### Type locality.

El Mogote, Baja California, Mexico.

##### Distribution.

Gulf of California, Mexico; shallow waters.

##### Remarks.

Although this species occurs in an area with a long history of marine research, it is still little known, and the only study focused on this species is the original description by Carlgren (1951). The species description is based only on one individual and therefore knowledge is quite limited and morphological variation is not known to date. Thus, this species still lacks taxonomic confirmation as well as other studies.

##### Type material.

Smithsonian National Museum of Natural History – USNM 49454 (Holotype).

#### 33 Pachycerianthus johnsoni

Taxon classificationAnimaliaSpirulariaCerianthidae

(Torrey & Kleeburger, 1909)

32B0112D-2ED9-57C1-839C-2870066F9530

http://zoobank.org/789ED1B7-5FEF-49D4-937E-4B20740A295E

Cerianthus johnsoni Torrey and Kleeburger, 1909: 116, 119, 123–125; Pax 1910: 167; Pei 1998: 181
Pachycerianthus johnsoni : McMurrich 1910: 11; Carlgren 1912a: 44–47; Arai 1965: 205, 210; Arai 1971: 1679; 1680; Carter 1995: 6; Stampar et al. 2014b: 350, 352

##### Type locality.

Los Angeles, East Pacific, United States of America.

##### Distribution.

Only known from shallow water at the type locality.

##### Remarks.

This is another species described from the United States’ Pacific Coast by Torrey and Kleeburger (1909) with a relatively good amount of detail; like *P. insignis*, there have been no more subsequent detailed or comparative studies. Arai (1971) has been the only author that has mentioned the morphological characters of this species after the original description, but even this characterization was based on the characters listed in the original description. The taxonomic status of this species is doubtful.

##### Type material.

Not found in this study, but the original description provided a graphic representation.

#### 34 Pachycerianthus longistriatus

Taxon classificationAnimaliaSpirulariaCerianthidae

Carter, 1995

97CF18FE-44E6-55D6-9076-CDC11C959027

http://zoobank.org/96D33C91-AA78-42A2-918D-C9CB9B6B3EB8

Pachycerianthus longistriatus Carter, 1995: 3–5; Stampar et al. 2014b: 350, 352

##### Type locality.

off Port Jackson, Sydney harbor, Australia.

##### Distribution.

Sydney Harbor, Australia; 5–10 m depth.

##### Remarks.

As mentioned for *P. delwynae*, the taxonomic status between the two Australian species, *P. delwynae* and *P. longistriatus*, is not clear. Both were described from a very restricted area and the morphological variation between them is very subtle. There is a need for a more detailed study approach to understand the differences between these two currently valid species.

##### Type material.

Australian Museum – AM G15402 (Holotype).

#### 35 Pachycerianthus magnus

Taxon classificationAnimaliaSpirulariaCerianthidae

(Nakamoto, 1919)

B5F04FCC-FA2C-5637-9D64-4CFF1BF80499

http://zoobank.org/909B29CC-61CA-4766-A000-73FB6FFAFB18

Cerianthus magnus Nakamoto, 1919: 118–120
Pachycerianthus magnus : Uchida 1979: 186–189; Carter 1995: 6; Uchida and Soyama 2001: 125, 151, 152; Molodtsova 2004b: 261; Stampar et al. 2014a: 2; Stampar et al. 2014c: 350, 352; Stampar et al. 2019: 1–9

##### Type locality.

south of Jogashima, Sagami Bay, Miura, Kanagawa, Japan (at 1100 m depth).

##### Distribution.

Japan and China, shallow to deep waters.

##### Remarks.

The description of *Cerianthus magnus* by Nakamoto (1919) is quite adequate, but still very simple. Nevertheless, the author presented a scheme of the mesenteries, and two photos of preserved and dissected material, which allows an adequate comparison with other species. Uchida (1979) moved this species to the genus *Pachycerianthus* and performed a very detailed redescription of the species based on specimens from Sagami Bay, Japan. This species occurs in an area with several other species of Ceriantharia but is apparently consistent with regard to its taxonomy. The co-occurrence of these species in Sagami Bay may be relevant in an evolutionary context with a focus on environmental niche differentiation among Ceriantharia species.

##### Type material.

Not found in this study, but the original description provided a graphic representation.

#### 36 Pachycerianthus maua

Taxon classificationAnimaliaSpirulariaCerianthidae

(Carlgren, 1900)

ADA1C99B-341A-56D2-B85F-0810C60057F3

http://zoobank.org/7797F482-1430-400F-ABD3-7FB2E511A132

Cerianthus maua Carlgren, 1900: 27–29; Krempf 1905: 195; Pax 1909: 413; Pax 1910: 167; Schmidt 1972: 427, 433; Emig et al. 1972: 304–307
Cerianthus mana Fishelson 1970: 109
Pachycerianthus maua : Carlgren 1912b: 389–391; Arai 1971: 1680; Carter 1995: 5; Stampar et al. 2014b: 350, 352

##### Type locality.

Mkokotoni, Zanzibar, Tanzania.

##### Distribution.

Indian Ocean (Mozambique, Madagascar, and Tanzania) and Aden Gulf (Djibouti) and Red Sea (Egypt and Saudi Arabia), shallow waters.

##### Remarks.

This species was described by Carlgren (1900), who subsequently moved this species to the genus *Pachycerianthus* and added some comments on its morphology (Carlgren 1912b). Krempf (1905) recorded two specimens from Djibouti, but he did not study anatomical characteristics. Much later, Fishelson (1970) recorded a great number of specimens from Eilat, Israel. However, again, the author failed to mention any anatomical characters of the specimens, and the figure of the presented specimen is quite inconsistent with the description of Carlgren (1900) or with specimens analyzed from Mozambique (S. Stampar pers. obs.). Thus, the identification of the Red Sea specimens may be misleading, and these Red Sea specimens could be classified either as another species already known to the region (perhaps *Cerianthus medusula*) or as an undescribed species.

##### Type material.

Not found in this study, but the original description provided a graphic representation.

#### 37 Pachycerianthus monostichus

Taxon classificationAnimaliaSpirulariaCerianthidae

McMurrich, 1910

E92CD37A-DA3D-513D-94A2-405080F5ADFB

http://zoobank.org/CDDC2972-281C-488C-84C4-BD5D468ADFA1

Pachycerianthus monostichus McMurrich, 1910: 38–39; Carlgren 1912a: 44–47; Arai 1971: 1680; van Soest 1979: 118; Carter 1995: 6; den Hartog 1997: 352; Stampar et al. 2014b: 350 352

##### Type locality.

Ambon, Maluku, Indonesia.

##### Distribution.

Only known from shallow water at the type locality.

##### Remarks.

This species was described by McMurrich (1910) based on two specimens from Ambon Island, Indonesia. The information presented by the author is quite suitable for characterization of the species, especially as the mesentery organization is quite conspicuous. No further relevant information on this species is available to date.

##### Type material.

Not found in this study, but the original description provided a graphic representation.

#### 38 Pachycerianthus multiplicatus

Taxon classificationAnimaliaSpirulariaCerianthidae

Carlgren, 1912

C5E81A52-5329-5FB9-894A-D7F35E5010F9

http://zoobank.org/1D9265CA-F705-4693-B955-D17D0A63500A

Cerianthus membranaceus : Lütken 1889: 362
Cerianthus danielssen : Levinsen 1893: 397; Carlgren 1896: 174
Pachycerianthus multiplicatus Carlgren, 1912a: 5–11; Carlgren 1931: 8–9; Carlgren 1940: 9–12; Carlgren 1942: 70–71; Carlgren 1945: 68–70; Schmidt 1972: 427, 432–433; Arai 1971: 1689; Keegan and Könnecker 1973: 257 Mariscal et al. 1977: 395; Manuel 1981: 64, 67; Picton 1985: 485; McFarlane 1988: 365–370; Carter 1995: 6; Molodtsova 2000: 12, 15, 17; Jonsson et al. 2001: 189–195; Stampar et al. 2014b: 350, 352
(?) Pachycerianthus multiplicatus: Çinar et al. 2014: 684

##### Type locality.

Two areas are mentioned – Kattegat Strait and Trondheim, Norway (not specified)

##### Distribution.

North, Inner, Celtic, Irish and Norwegian Seas, Gulf of Biscay, at < 130 m depth.

**Remarks.**Levinsen (1893) described this species as *Cerianthus danielssen*, however, this description was incomplete and did not meet the minimum characterization requirements for a cerianthid species. Thus, Carlgren (1912a) proposed the new name *P. multiplicatus*, while giving a detailed description of this species. Carlgren (1912b) included several records in the region as well as some biological aspects. Nyholm (1943) gave a detailed study of the life cycle of the species, including information on reproductive seasons and also on larval development (a modified planula). This is a very interesting species for ecological studies, as several reports have mentioned clusters of individuals in different regions (e.g., Jonsson et al. 2001). There is still doubt about the true distribution of the species as individuals recorded from the coast of France and Spain have never been studied in detail.

##### Type material.

(?) Lund Museum of Zoology (MZLU) - 6570 (syntype), but not formally designated in description.

#### 39 Pachycerianthus nobilis

Taxon classificationAnimaliaSpirulariaCerianthidae

(Haddon & Shackleton, 1893)

83500BE5-4C74-54F1-9756-ACF1152156E1

http://zoobank.org/7EE6D6AD-FAF0-447C-A609-D2D03A083096

Cerianthus nobilis Haddon and Shackleton, 1893: 116, 118; Carlgren 1896: 174; Haddon 1898: 400–401; Pax 1910: 167
Pachycerianthus nobilis : Molodtsova 2000: 19; Molodtsova 2007: 133; Stampar et al. 2014b: 350, 352

##### Type locality.

Thursday Island, Queensland, Australia.

##### Distribution.

Queensland and Northern Territory, Australia, New Caledonia, shallow waters.

##### Remarks.

A large species originally described from northeastern Australia as *Cerianthus nobilis*. This description is very simple and was based only on external characters and there have been no further studies based on specimens from this area. Molodtsova (2000, 2007) correctly suggest that this species does not belong to the genus *Cerianthus*, but to the genus *Pachycerianthus*. The relation with two species described by Carter (1995) (*P. delwynae* and *P. longistriatus*) is completely unknown, however, there is the possibility that all three species are, in fact, a single one, based on their overlapping morphological characters.

##### Type material.

Museum of Zoology (University of Cambridge) – I.33575.A-B (holotype).

#### 40 Pachycerianthus schlenzae

Taxon classificationAnimaliaSpirulariaCerianthidae

Stampar, Morandini & Silveira, 2014

584E45C7-6EF0-5D13-B0C2-47601794EC8B

http://zoobank.org/7D2022C6-CE8E-4FC4-B54D-39ED0006FA7F

Pachycerianthus sp. Vieira and Stampar 2014: 365, 367–368, 370–371
Pachycerianthus schlenzae Stampar et al., 2014b: 343–354

##### Type locality.

off Guarapari, Espírito Santo state, Brazil.

##### Distribution.

Brazil, from Bahia to Espírito Santo states (Abrolhos Bank and Royal Charlotte Bank), at 5–10 m depth.

##### Remarks.

This species was recently described based on a study of several specimens from the central area of the Brazilian coast, where it is an endemic occurring along a coastline of approximately 500 km length. Some aspects of external morphology are similar to those of *P. curacaoensis* and may reflect a correlated evolutionary history between the two species. Although Stampar et al. (2014b) presented some biological information mainly related to the reproduction seasons, little is known about the ecology and biology of this species. The species is endangered as its range suffers from high levels of anthropogenic pressure (Miranda and Marques, 2016), which could result in the loss of its habitat.

##### Type material.

Museu de Zoologia, Universidade de São Paulo (MZSP) – 1949 (Holotype).

#### 41 Pachycerianthus solitarius

Taxon classificationAnimaliaSpirulariaCerianthidae

(Rapp, 1829)

4E557877-B62E-5666-8AAA-4E4D2402709A

http://zoobank.org/DDC053BF-7A9C-4CB8-9126-FE701530AB39

Tubularia solitaria Rapp, 1829a: 656–658; Rapp 1829b: 48–49
(?) Cereus cupreus Ilmoni, 1830: 689–699; Ilmoni 1831: 123
Cerianthus brerae Delle Chiaje, 1841: 136
Edwardsia vestita Forbes, 1843: 42; Milne-Edwards 1857: 286
Cerianthus membranaceus : Sars 1857: 28–32; Heller 1868: 20, 79
Cerianthus solitarius : Andres 1881: 332; Andres 1883: 345–346; Fischer 1887: 384–385, 432, 437; Fischer 1889: 254, 264, 265; Faurot 1895: 221; van Beneden 1897: 138; Child 1903a: 239–260; Child 1903b: 8–10; Child 1904a: 70–71; Child 1904b: 266–284; Gravier 1904: 283; Rioja and Martin 1906: 280; Cerfontaine 1909: 699–700; McMurrich 1910: 17–18; Pax 1910: 166; Mello-Leitão 1919: 35–36, 39; van Beneden 1924: 29, 89–96; Torelli 1961: 17–28
Pachycerianthus solitarius : Carlgren 1912a: 367–387; Carlgren 1912b: 44–47; Carlgren 1927: 443; Panikkar 1936: 259; Pax and Müller 1962: 110; Torelli 1963: 175–177; Naumov 1968: 73; Arai 1971: 1680; Uchida 1979: 188; Williams 1981: 350; Ates 1985: 230–231; Carter 1995: 6; Molotsova 2003: 250, 252–253; Çinar et al. 2014: 684
(?) Pachycerianthus solitarius: Kisseleva 1975: 1595–1596; Wirtz et al. 2003: 115, 116
Cerianthus bicyclus Torelli, 1961: 17–28

##### Type locality.

off Languedoc coast, France.

##### Distribution.

Mediterranean Sea, Azores, and (?) Black Sea; shallow waters.

##### Remarks.

After *Cerianthus membranaceus*, this was the second species to be formally described in Ceriantharia. It was first described as an unclassified polyp with some similarities with Hydrozoa and Anthozoa (Rapp 1829a). Later, it was only characterized as a Ceriantharia by Sars (1857). This species was widely studied by Child (1903a, 1903b, 1904a, 1904b) in a series of experimental studies related to asexual reproduction, behavior, and regeneration of polyps. Carlgren (1912b) presented a detailed redescription of this species and moved it to the genus *Pachycerianthus*. He described several abnormalities of the species’ anatomy (Carlgren 1912a), which were attributed to asexual reproduction events that are uncommon in Ceriantharia. After this, several authors reported this species from various regions, including the Black Sea (Kiseleva 1975). If *P. solitarius* occurs in the Black Sea, it would show a great tolerance to brackish water. Therefore, specimens recorded in the Black Sea may not be of the same species that inhabits the Mediterranean Sea. However, there are no available specimens for comparison. Wirtz et al. (2003) recorded this species in the Azores, however a more detailed study is needed to understand if this species occurs outside the Mediterranean Sea, or whether the Azores specimens belong to *P. solitarius*.

##### Type material.

Not found in this study.

**Family Botrucnidiferidae Carlgren, 1912**

Number of valid taxa: two genera and four species.

#### Genus Botruanthus

Taxon classificationAnimaliaSpirulariaCerianthidae

McMurrich, 1910

6F953DE3-C93B-579B-B8E9-BF8C09204236

Table 5

##### Type species.

*Botruanthus benedeni* (Torrey & Kleeberger, 1909)

Number of valid species: 2

**Table 5.** Comparison of anatomical features of *Botruanthus* species (after Stampar et al. 2016a).

	*B. benedeni*	*B. mexicanus*
**Marginal tentacles**	Up to 90–100	Up to 40–60
**Directive labial tentacle**	Present	Present
**Arrangement of labial tentacles**	(1)321.3213.3213	(2)314.2314.2314.2314
**Actinopharynx**	1/3 – 1/4 of gastric cavity	1/5–1/4 of gastric cavity
**Oral disc**	1.1–1.3 cm	0.5–0.7 cm
**Siphonoglyph**	Broad, 8 mesenteries attached	Narrow, 2 mesenteries attached
**Directive mesenteries**	>Actinopharynx (= size of Actinopharynx)	> Actinopharynx
**P2**	Long, almost to aboral pole (> 2/3 of gastric cavity)	Short (<1/3 of gastric cavity)
**P3**	Short (1/3 of P2)	Short (~P2)
**M1**	To aboral pore	Almost to aboral pore
**M3**	Almost to aboral pore	Short, 1/2 of M1
**Cnido-glandular tract at fertile mesenteries of first quartets**	Present	Present
**Craspedion tract at fertile mesenteries**	5/7–8/9	8/9
**Cnido-glandular tract at B**	< ½	3/4
**Craspedonemes of craspedion at fertile mesenteries**	Sometimes present	Sometimes present
**Botrucnidae**	Rare in m and B, absent in M and b mesenteries	Very abundant (4-5 groups) in M and m, absent in B and b mesenteries

#### 42 Botruanthus benedeni

Taxon classificationAnimaliaSpirulariaCerianthidae

(Torrey & Kleeberger, 1909)

470AB94A-8580-55EF-ACBE-B9092DDB7347

http://zoobank.org/B663D1F1-44EB-4EBC-8644-729518D0104A

Pachycerianthus benedeni Roule, 1904: 708–710
Cerianthus benedeni : Torrey and Kleeberger 1909: 115, 119, 120–123, 125; Pax 1910: 167;
Botryanthus benedeni : McMurrich 1910: 11; Torelli 1932: 9
Botruanthus benedeni : Carlgren 1912a: 44–47; Leloup 1932: 17; Carlgren 1951: 431, 433–435; Arai 1965: 205; den Hartog 1977: 211, 233, 236–237; Molodtsova 2001c: 1027, 1033–1035; Fautin et al. 2007: 551–552, 567–569; Stampar et al. 2016b: 1; 5

##### Type locality.

San Diego Bay, California, United States of America.

##### Distribution.

California (United States of America), Baja California (Mexico) and Galapagos Islands (Ecuador), shallow waters.

##### Remarks.

This species was described based on a study of a single specimen. This species (and genus) is characterized by possessing wart-like structures (cnidorages) organized in bunches (botrucnids) in the mesenterial filaments. Except for these structures, the anatomy is very similar to species of the genus *Pachycerianthus*. The holotype is not available, and we therefore here designate a neotype collected from the same region by Charles Cutress in 1955 (NMNH 49400). This specimen was studied by Stampar et al. (2017) (erroneously referred to as “holotype”) and its characters are consistent with those in the original description. Because of its importance as type species of the genus *Botruanthus*, the neotype designation for this species is justified. This is a poorly studied genus and there is no biological or ecological information about this species.

##### Type material.

Smithsonian National Museum of Natural History (USNM) – 49400 (neotype).

#### 43 Botruanthus mexicanus

Taxon classificationAnimaliaSpirulariaCerianthidae

Stampar, González-Muñoz & Morandini, 2017

6921C670-26FA-59EE-9E2C-8F69E64CD145

http://zoobank.org/61DE065D-E0EB-4BE4-A283-E7EADFCD7262

Botruanthus mexicanus Stampar et al., 2017: 113–118

##### Type locality.

off Veracruz, Mexico.

##### Distribution.

Gulf of Mexico, intertidal to shallow waters.

##### Remarks.

This species was recently described by specimens from the intertidal zone in reefs of Central Mexico in the Gulf of Mexico. Morphological characterization is quite easy, as the number of anatomical characters allow its distinction in relation to *B. benedeni*. There have been no studies on ecological or biological aspects of this species.

##### Type material.

Museu de Zoologia da Universidade de São Paulo; MZUSP 002757 (Holotype).

#### Genus Botrucnidifer

Taxon classificationAnimaliaSpirulariaCerianthidae

Carlgren, 1912

82E59A75-20BD-5F65-BC3D-B5E42CF85248

Table 6

##### Type species.

*Botrucnidifer norvegicus* Carlgren, 1912

Number of valid species: two

**Table 6.** Comparison of anatomical features of *Botrucnidifer* species.

	*B. novergicus*	*B. shtokmani*
**Marginal tentacles**	Up to 17	72
**Directive labial tentacle**	Present	Absent
**Arrangement of labial tentacles**	(1)431.3231.3231	(0)230.2024.3123.3142
**Actinopharynx**	1/4 – 1/5 of gastric cavity	1/3 of gastric cavity
**Oral disc**	0.3 cm	1.5 cm
**Siphonoglyph**	Narrow, 2 mesenteries attached	Narrow, 4 mesenteries attached
**Directive mesenteries**	>Actinopharynx	= Actinopharynx
**P2**	Long, almost to aboral pole (> 4/5 of gastric cavity)	Regular (<2/3 of gastric cavity)
**P3**	Long (2/3 of P2)	Short, 1/2 of P2
**M1**	Almost to aboral pore	= P2
**M3**	Almost to aboral pore (3/4 of M1)	Long, 3/4 of P2
**Cnido-glandular tract at fertile mesenteries of first quartets**	Present	Present
**Craspedion tract at fertile mesenteries**	¾	1/2 – 3/4
**Cnido-glandular tract at B**	Present	Present
**Botrucnidae**	Only in M mesenteries	Only in B/b mesenteries

#### 44 Botrucnidifer norvegicus

Taxon classificationAnimaliaSpirulariaCerianthidae

Carlgren, 1912

DFD2C112-B37A-5AE0-B89F-370F971443A3

http://zoobank.org/5D96E928-EB37-4362-ADD0-F20293438F44

Botrucnidifer novergicus Carlgren, 1912a: 30–34; Carlgren 1931: 10; Leloup 1932: 16–18; Carlgren 1940: 6,10,14–15; Carlgren 1942: 70–71; Carlgren 1945: 72; Nair 1949: 245; den Hartog 1977:136; Molodtsova 2000: 14–17; Molodtsova 2001c: 1027, 1033–1036; Molodtsova 2004a: 292–293, 295–296; Stampar et al. 2016c: 2,4; Ceriello et al. 2019: 2017–2020

##### Type locality.

Trondheimfjord, Trondheim, Norway.

##### Distribution.

Norwegian Sea, at 50–700 m depth.

##### Remarks.

This species was described by Carlgren (1912a) based on specimens from Trondheim Fjord, Norway. These are small ceriantharians (up to 4 cm long) with an expansion of the cnidoglandular tract and some botrucnidae (= cnidoragae) at the end of some mesenteries. Although the description is fairly comprehensive, knowledge of this species is limited. Other authors cite only some of the species characteristics or have reported occurrences in areas that look similar (e.g., Molodtsova 2004a). Recently, Ceriello et al. (2019) reported on the coloniality of this species, which is a newly discovered trait among Ceriantharia. This species is important in the discussion on the homology of morphological characters, particularly in relation to mesenterial structures.

##### Type material.

Lund Museum of Zoology (MZLU) – L898/3051 and Marine invertebrate collection Norwegian University of Science and Technology University Museum (NTNU) – 40499 (syntype).

#### 45 Botrucnidifer shtokmani

Taxon classificationAnimaliaSpirulariaCerianthidae

Molodtsova, 2001

BBE8D936-DF04-5515-821F-F462ADB959D1

http://zoobank.org/2B2E3575-21EB-4B85-80AA-817B45BB89A1

Botrucnidifer shtokmani Molodtsova, 2001a: 773; Molodtsova 2001c: 1027–1036; Molodtsova et al. 2011: 1

##### Type locality.

off Namibia coast (southeast Atlantic), at 130–350 m depth.

##### Distribution.

Only known from deep water at the type locality.

##### Remarks.

This species was described based on dredged specimens from off the Namibian coast. This is the second species of this genus that has been sampled beyond conventional SCUBA diving depths. The description of this species (in Russian) is very detailed and addresses all the necessary characters. As discussed by Molodtsova (2001c) some larval forms of this family are recognized from this area, however the link between larval and adult stages is only possible based on molecular or developmental approaches (Nyholm 1943; Stampar et al. 2015c).

##### Type material.

Zoological Museum of Moscow University – ZMMU EC-100 (holotype).

**Order Penicillaria den Hartog, 1977**

Number of valid taxa: one family, two genera, and nine species

**Family Arachnactidae McMurrich, 1910**

Number of valid taxa: two genera, and nine species

#### Genus Arachnanthus

Taxon classificationAnimaliaSpirulariaCerianthidae

Carlgren, 1912

AB7CDF4A-F3DC-59F8-BEB5-C827F2A78FBF

Table 7

##### Type species.

*Arachnanthus oligopodus* (Cerfontaine, 1891)

Number of valid species: Five

**Table 7.** Comparison of anatomical features of *Arachnanthus* species (after Stampar et al. 2018).

	*A. australiae*	*A. bockii*	*A. oligopodus*	*A. sarsii*	*A. lilith*
**Marginal tentacles**	Up to 40	Up to 30	~20	Up to 35	Up to 24
**Arrangement of labial tentacles**	(0)1.11.11.11.11	(0)1.11.11.11.11(?)	(0)1.11.11.11.11	(0)1.11.11.11.11	(0)3.12.31.23.23.12
**Length of actinopharynx**	~2/3 of gastric cavity	~1/2 of gastric cavity	~1/2 of gastric cavity	~1/2 of gastric cavity	>1/2 of gastric cavity
**Hyposulcus**	~1/2 size of stomodeum	~1/2 size of stomodeum	~2X size of stomodeum	< size of stomodeum	= size of stomodeum
**Oral disc diameter**	~0.7 cm	–	–	~1 cm	0.5 cm
**Mesentery attachment to actinopharynx**	Broad, 12 mesenteries attached	Broad, 12 mesenteries attached	Narrow, 4 mesenteries attached	Broad, 6 mesenteries attached	Broad, 8 mesenteries attached
**Directive mesenteries**	= length of Actinopharynx	< length of Actinopharynx	> length of Actinopharynx	< length of Actinopharynx	< length of Actinopharynx
**P(C)2**	Short, 1/2 of gastric cavity	Very short, 1/4 of gastric cavity	Short, 1/2 of gastric cavity	Long, 3/4 of gastric cavity	Long, 6/7 of gastric cavity, almost to aboral pole
**P(C)3**	Very short, <1/4 of gastric cavity	Very short, <1/4 of gastric cavity	Short, ~1/2 of gastric cavity	Short, ~1/3 of gastric cavity	Short, 1/3 of gastric cavity
**M1**	Almost to aboral pore	Almost to aboral pore	To aboral pore	Almost to aboral pore	To aboral pore
**M3**	4/5 of gastric cavity	Almost to aboral pore	1/5 of gastric cavity	Almost to aboral pore	3/4 of gastric cavity
**Cnido-glandular tract of fertile mesenteries**	Present (short?)	Present (short?)	Present	Present	Present
**Cnido-glandular tract of B**	Present (short?)	Present (short?)	Present (short?)	Present (short)	Present (short)
**Acontioids**	Only in M1, M2 and M3	Only in M1, M2 and M3	Only in M1	Only in M1, M2 and M3	Only in M3 and M4

#### 46 Arachnanthus australiae

Taxon classificationAnimaliaSpirulariaCerianthidae

Carlgren, 1937

3CF110AF-D3D1-5540-A925-AA9BF33F3043

http://zoobank.org/249FDE31-6249-45B8-8930-D87A827BC87B

Arachnanthus australiae Carlgren, 1937: 177–180; den Hartog 1977: 235; Fautin et al. 2007: 570; Stampar et al. 2018: 3,8

##### Type locality.

Low Isles, Queensland, Australia.

##### Distribution.

Queensland, Australia, shallow waters.

##### Remarks.

Carlgren (1937) described this species from northeastern Australia and this is the only study so far on this species. Although the description is adequate, it does not include detailed information. In general, the Australian coast is vastly understudied regarding Ceriantharia species diversity. The taxonomic status of *A. australiae* in relation to *Arachnanthus bockii* remains to be studied in detail.

##### Type material.

Natural History Museum (London); NHMUK – 1954.6.25.47 (holotype).

#### 47 Arachnanthus bockii

Taxon classificationAnimaliaSpirulariaCerianthidae

Carlgren, 1924

31DB6E9D-0D57-586B-BAA1-DF7D0C2FD6CF

http://zoobank.org/357544F3-9111-420C-B6AD-B9A446BB9451

Arachnanthus bockii Carlgren, 1924: 193–195; den Hartog 1977: 235

##### Type locality.

Viti Levu, Fiji.

##### Distribution.

Only known from shallow water at the type locality.

##### Remarks.

This is another species with little information, except for the morphological description. There are some characters in Carlgren’s (1924) description that allows distinction of this species in comparison to *Arachnanthus australiae*, however, the reduced number of specimens may be a problem to understand the intraspecific variation of these characters.

##### Type material.

Not found in this study, but the original description provided a graphic representation.

#### 48 Arachnanthus oligopodus

Taxon classificationAnimaliaSpirulariaCerianthidae

(Cerfontaine, 1891)

C3D3C37D-D009-5735-9EC6-80D8A30238C2

http://zoobank.org/7B2B79CA-DC0A-4E14-9ED0-06F2AB20C245

Cerianthus oligopodus Cerfontaine, 1891a: 32–38; Carlgren 1895: 284; van Beneden 1897:140; Gravier 1904: 286; Cerfontaine 1909: 653–707; McMurrich 1910a: 165; Pax 1910: 166; Mello-Leitão 1919: 36, 39; van Beneden 1924: 12, 20, 30, 45, 92, 97, 98; Torelli 1932:12; Torelli 1961: 17–28
Pachycerianthus oligopodus : McMurrich 1910b: 11–13
Arachnanthus oligopodus : Carlgren 1912a: 367–388; Carlgren 1912b: 44–47; Panikkar 1947: 243; Leloup 1960: 2; den Hartog 1977: 235; Vafidis and Koukouras 1998: 123–124; Molodtsova 2003: 253; Çinar et al. 2014: 677, 683, 687, 688; Rastorgueff et al. 2015: 142, 148

##### Type locality.

Italian Coast, Mediterranean Sea (not specified in detail).

##### Distribution.

Mediterranean Sea, shallow waters and caves.

##### Remarks.

*Arachnanthus oligopodus* was initially described as a species of the genus *Cerianthus* by Cerfontaine (1891a), and was moved to the genus *Arachnanthus* by Carlgren (1912b). This is a very common species in several areas of the Mediterranean Sea, especially on the Italian Coast (Carlgren 1924; Torelli 1961). This species has a number of descriptions with appropriate levels of detail (e.g., Cerfontaine 1909; Carlgren 1912a). However, knowledge of the species is still incipient. Biological aspects, especially on the life cycle, are still quite unknown.

##### Type material.

Not found in this study.

#### 49 Arachnanthus lilith

Taxon classificationAnimaliaSpirulariaCerianthidae

Stampar & El Didi in Stampar et al. 2018

C79DB8EA-73BB-58EB-BA8B-F37BB639BA58

http://zoobank.org/fc381c67-9db8-4280-9c9c-00dbd04f7d56

Arachnanthus lilith Stampar and El Didi in Stampar et al. 2018: 1–7

##### Type locality.

island near Jaz’air Sila, Saudi Arabia.

##### Distribution.

Red Sea, shallow waters.

##### Remarks.

This species was recently described from shallow Saudi Arabian waters of the Red Sea. Morphological characterization was based on internal anatomy and there have been no studies on ecological or biological aspects of this species yet.

##### Type material.

Florida Museum of Natural History – FLMNH UF9168 (holotype).

#### 50 Arachnanthus sarsi

Taxon classificationAnimaliaSpirulariaCerianthidae

Carlgren, 1912

C7E918DA-5C4A-5678-9D1A-0EF53F8167F3

http://zoobank.org/04EE6C07-87AA-4137-8391-D6FE93024EFB

Arachnanthus sarsi Carlgren, 1912a: 27–30; Carlgren 1942: 70–71; Nair 1949: 243; Picton and Manuel 1985: 343–349; Picton 1985: 485–486; Molodtsova 2000: 15, 17; Molodtsova 2003: 251; Stampar et al. 2015c: 2164
Arachnanthus sarsii : Carlgren 1931: 9–10; Carlgren 1940: 6, 11, 13, 15

##### Type locality.

Röberg Indalbay, Trondheim, Norway.

##### Distribution.

North Sea, at 10–200 m depth.

##### Remarks.

This species is rather common in some areas of Great Britain and Scotland and there are two detailed descriptions; the original (Carlgren 1912b) and a redescription (Picton and Manuel 1985). The life cycle has been inferred from the occurrence of larvae named as *Arachnactis albida* (Picton and Manuel 1985), but further study is needed to understand the relationship in detail. Not much is known about ecological aspects of this species and this should be a very interesting field of study.

##### Type material.

Swedish Museum of Natural History (Naturhistoriska riksmuseet) – NRM 134778 (Holotype).

#### Genus Isarachnanthus

Taxon classificationAnimaliaSpirulariaCerianthidae

Carlgren, 1924

176A5DEE-6D84-5820-86E8-4E52984F2642

Table 8

##### Type species.

*Isarachnanthus maderensis* (Johnson, 1861)

Number of valid species: 4

**Table 8.** Comparison of anatomical features of *Isarachnanthus* species.

	*I. bandanensis*	*I. maderensis*	*I. nocturnus*	*I. panamensis*
**Marginal tentacles**	Up to 40	Up to 42	Up to 60	Up to 32
**Arrangement of labial tentacles**	(3)413.4242.4312.4312	(1)1.11.11.11.11	(1)2.12.12.12.12	(2)431.4231.4231
**Length of actinopharynx**	~1/4 of gastric cavity	~2/5 of gastric cavity	~2/5 of gastric cavity	~1/2 to 1/3 of gastric cavity
**Hyposulcus**	~2/3 size of stomodeum	= size of stomodeum	= size of stomodeum	= size of stomodeum
**Oral disc diameter**	~2cm	2 cm	3.5 cm	~0.5 cm
**Mesentery attached to siphonoglyph**	Broad, 18 mesenteries attached	Broad, 10 mesenteries attached	Broad, 12-14 mesenteries attached	Broad, 16 mesenteries attached
**Directive mesenteries**	= length of Actinopharynx	> length of Actinopharynx	> length of Actinopharynx	>length of Actinopharynx
**P(C)2**	Long, 3/4 of gastric cavity	Short, 1/3 of gastric cavity	Short, 1/3 of gastric cavity	Long, 3/4 of gastric cavity
**P(C)3**	Very short, <1/8 of gastric cavity	Very short, ~1/6 of gastric cavity	Very short, ~1/5 of gastric cavity	Short, ~1/5 of gastric cavity
**M1**	Almost to aboral pore	Almost to aboral pore	Almost to aboral pore	Reach aboral pore
**M3**	Almost to aboral pore	Almost to aboral pore	Almost to aboral pore	Reach aboral pore
**Cnido-glandular tract of fertile mesenteries**	Present (short?)	Present	Present	Present (short?)
**Cnido-glandular tract of B**	Present (short?)	Present (short)	Present (short)	Present (short?)
**Acontioids**	Only in M1- M4	Only in M1-M6	M1-M3 (sometimes in M4 and M5)	Only in M1- M5 or absent

#### 51 Isarachnanthus bandanensis

Taxon classificationAnimaliaSpirulariaCerianthidae

Carlgren, 1924

74D105BD-66CF-5354-B9D0-89871EF96434

http://zoobank.org/66c30a7f-149e-4c92-8a8d-c7a63fe71194

Isarachnanthus bandanensis Carlgren, 1924: 187–190, 195; Cutress 1977: 145; den Hartog 1977: 235; Cutress and Arneson 1987: 54, 56–58; den Hartog 1997: 352; Stampar et al. 2012: 1–2, 5–9.

##### Type locality.

Neira, Banda Island, Indonesia.

##### Distribution.

Indonesia, French Polynesia, and Hawaii (USA), shallow waters.

##### Remarks.

This species was described based on two specimens from the Banda Islands, Indonesia. The diagram of mesenteries, part of cnidome, and tentacle organization are present in the original description, however, there are some evident similarities in relation to *Isarachnanthus panamensis*. Furthermore, unpublished molecular data indicate similarity between these two species and studies on this clade should be prioritized.

##### Type material.

Zoological Museum of Amsterdam (now Naturalis Biodiversity Center, Leiden) – (ZMA.COEL.000209 – Lectotype/ ZMA.COEL.000210 – Paralectotype).

#### 52 Isarachnanthus maderensis

Taxon classificationAnimaliaSpirulariaCerianthidae

(Johnson, 1861)

CCEB0788-9093-5718-908B-43818509081D

http://zoobank.org/B6923781-7CEE-4F15-89C6-A35765626176

Saccanthus maderensis Johnson, 1861: 305–306; Andres 1883: 346
Cerianthus maderensis : Pax 1908: 262–263;
In part Cerianthus membranaceusPax 1908: 464–465, 497–498
Arachnanthus nocturnus : Ocaña et al. 2000b: 107; Wirtz et al. 2003: 114–116
Isarachnanthus cruzi Brito, 1986: 174–181
? Cerianthus sp. Torelli 1963: 714–715
Isarachnanthus maderensis : Molodtsova 2003: 249–253; Stampar et al. 2012: 1–9; Stampar and Morandini 2017: 689–693

##### Type locality.

Madeira Island, Portugal.

##### Distribution.

Madeira Island (Portugal), Ascension Island, Rocas Atoll (Brazil), Caribbean Sea, (?) Mediterranean Sea; at 2–30 m depth.

##### Remarks.

This species was described by Johnson (1861) from Madeira Island. However, the first detailed morphological characterization was presented by Brito (1986) (as *I. cruzi*). The delimitation of this species is quite complicated, as according to Stampar et al. (2012) only molecular data or morphometric data of the cnidome can be used to compare to other species of the genus. The distribution of this species is quite wide, from oceanic islands of the South Atlantic to the Caribbean Sea and the Mediterranean Sea (Stampar and Morandini 2017).

##### Type material.

Not found in this study.

#### 53 Isarachnanthus nocturnus

Taxon classificationAnimaliaSpirulariaCerianthidae

(den Hartog, 1977)

7BF4DA21-894B-569A-B2C5-D905ADAD6CDF

http://zoobank.org/07411A5F-9150-4FDD-9080-565F5C4A8D00

Cerianthus natans : Verrill 1901: 47
Ceriantheopsis sp. Pax 1924: 94, 118–120
Arachnanthus nocturnus den Hartog, 1977: 221–230; Cairns et al. 1986: 192–193; Uchida and Soyama 2001: 142, 150, 152 Wirtz et al. 2003: 115–116;
Isarachnanthus nocturnus : Molodtsova 2000: 15,17; Molodtsova 2003: 251–252; Stampar et al. 2012: 1–9
Isarachnanthus sp. Rodriguez et al. 2011: 51; 52, 54
Tessera gemmaria Goy, 1979: 288–289; Rodriguez et al. 2011: 51–55; Stampar et al. 2015c: 2162

##### Type locality.

Piscadera Bay, Curaçao, Dutch Caribbean.

##### Distribution.

Caribbean Sea, South Atlantic (Argentina; Brazil), at 1–20 m depth.

##### Remarks.

This species was described by den Hartog (1977) based on specimens from Curaçao. The specific epithet is related to the nocturnal behavior of this species. This is the most studied species of the genus, as the larval development has been described and the taxonomy has been reviewed with molecular data (Stampar et al. 2012, 2015c). Molodtsova (2003) argued that this species is only a synonym of *Isarachnanthus maderensis*, however, based on molecular and micrometric data (Stampar et al. 2012) it has been shown that these two species are distinct.

##### Type material.

Naturalis Biodiversity Center, Leiden (former Rijksmuseum van Natuurlijke Historie) – RMNH.COEL.11364 (Holotype).

#### 54 Isarachnanthus panamensis

Taxon classificationAnimaliaSpirulariaCerianthidae

Carlgren, 1924

A0E8CE8E-DE4A-5F0E-BCD1-24FF52B75F8D

http://zoobank.org/CB322F62-0E21-4157-9107-4279DA1E16A9

Isarachnanthus panamensis Carlgren, 1924: 190–193, 195; Carlgren 1940: 6, 11, 13–14; Molodtsova 2003: 251; Stampar et al. 2012: 1–2

##### Type locality.

Taboga, Panama (Pacific coast).

##### Distribution.

Only known from shallow water at the type locality

##### Remarks.

This species was described from the Panama coast based on three specimens. The description is also detailed, including two mesentery diagrams. Thus, variation in mesenterial organization is quite evident, especially in relation to the size of directive mesenteries. As discussed above, regarding *Isarachnanthus bandanensis*, these two species are very similar in terms of both morphological and molecular data and further studies are needed.

##### Type material.

Zoological Museum of Amsterdam (now Naturalis Biodiversity Center, Leiden) – ZMA.COEL .000211) (holotype).

### Key to species

* Species with limited information on their anatomy, therefore key must be used with caution.

**Table:** 

1a	Ceriantharia with mesenteries organized in doublets (Spirularia)	**2**
1b	Ceriantharia with mesenteries organized in quartets (Penicillaria)	**16**
2a	Ceriantharia with cnidorage (botrucnidae)	**3**
2b	Ceriantharia without cnidorage (botrucnidae)	**6**
3a	Cnidorage on appendages united as botrucnidae	**4**
3b	Cnidorage over mesenteries	**5**
4a	P-mesenteries (P2) and M-mesenteries (M3) long, almost to aboral pore	***Botruanthus benedeni* (Torrey & Kleeburger, 1909)**
4b	P-mesenteries (P2) and M-mesenteries (M3) short, 1/2 to 1/3 of gastric cavity	***Botruanthus mexicanus* Stampar, González-Muñoz & Morandini, 2016**
5a	Directive mesenteries much longer than hyposulcus	***Botrucnidifer novergicus* Carlgren, 1912**
5b	Directive mesenteries shorter or equal than hyposulcus	***Botrucnidifer shtokmani* Molodtsova, 2001**
6a	Ceriantharia with all mesenteries except directives fertile	**7**
6b	Ceriantharia with second couple of protomesenteries (P) short and sterile	**8**
6c	Ceriantharia with second couple of protomesenteries (P) long and fertile, mesenteries in quartets m, B, M, b	**11**
6d	Ceriantharia with second couple of protomesenteries (P) long and fertile, mesenteries in quartets M, B, m, b	**12**
7a	Directive mesenteries of the same length as protomesenteries 3 (P3)	***Ceriantheomorphe brasiliensis* (Mello-Leitão, 1919)**
7b	Directive mesenteries shorter than protomesenteries 3 (P3)	***Ceriantheomorphe ambonensis* (Kwietniewski, 1898)**
7c	Directive mesenteries longer than protomesenteries 3 (P3)	***Ceriantheomorphe adelita* Lopes, Morandini & Stampar in Lopes et al., 2019**
8a	Number of marginal tentacles – less than 90	**9**
8b	Number of marginal tentacles – more than 115	**10**
9a	Metamesenteries 2 (M2) longer than ¾ of metamesenteries 1 (M1) and 6 mesenteries attached to siphonoglyph	***Pachycerianthus schlenzae* Stampar, Silveira & Morandini, 2014**
9b	Metamesenteries 2 (M2) longer than ¾ of metamesenteries 1 (M1) and more than 90 marginal tentacles	***Pachycerianthus johnsoni* (Torrey & Kleeburger, 1909)**
9c	Metamesenteries 2 (M2) longer than ¾ of metamesenteries 1 (M1) and less than 70 marginal tentacles	***Pachycerianthus fimbriatus* (Kwietniewski, 1898)**
9d	Metamesenteries 2 (M2) longer than half of metamesenteries 1 (M1) and 4 mesenteries attached to siphonoglyph	***Pachycerianthus curacaoensis* den Hartog, 1977**
9e	Metamesenteries 2 (M2) longer than metamesenteries 1 (M1), 6 mesenteries attached to siphonoglyph and directive labial tentacle present	***Pachycerianthus delwynae* Carter, 1995**
9f	Metamesenteries 2 (M2) longer than Metamesenteries 1 (M1) and 16 mesenteries attached to siphonoglyph	***Pachycerianthus aestuarii* (Torrey & Kleeburger, 1909)**
9g	Metamesenteries 2 (M2) and metamesenteries 1 (m1) longer than metamesenteries 1 (M1) and 8 mesenteries attached to siphonoglyph	***Pachycerianthus insignis* Carlgren, 1951**
9h	Metamesenteries 2 (M2) and metamesenteries 2 (m2) longer than metamesenteries 1 (M1) and 8 mesenteries attached to siphonoglyph	***Pachycerianthus monostichus* McMurrich, 1910**
9i	Metamesenteries 1 (m1) longer than ¼ of Metamesenteries 1 (M1) and 6 mesenteries attached to siphonoglyph	***Pachycerianthus solitarius* van Beneden, 1924**
10a	Metamesenteries 2 (M2) longer than metamesenteries 1 (M1) and metamesenteries 1 (m1) longer than than ¾ of M1	***Pachycerianthus borealis* Kingsley, 1904**
10b	Metamesenteries 2 (M2) longer than metamesenteries 1 (M1) and metamesenteries 1 (m1) longer than 1/3 of M1, labial directive tentacle present	***Pachycerianthus longistriatus* Carter, 1995**
10c	Metamesenteries 2 (M2) longer than ¾ of metamesenteries 1 (M1) and metamesenteries 1 (m1) longer than 1/3 of M1	***Pachycerianthus magnus* Uchida, 1979**
10d	Metamesenteries 2 (M2) longer than 1/4 of metamesenteries 1 (M1) and metamesenteries 1 (m1) longer than 1/3 of M1	***Pachycerianthus maua* Carlgren, 1900**
10e	Metamesenteries 2 (M2) longer than metamesenteries 1 (M1) and metamesenteries 1 (m1) longer than 1/3 of M1, labial directive tentacle absent	***Pachycerianthus multiplicatus* Carlgren, 1912**
10f	Polyp with more than 160 tentacles from Australia	***Pachycerianthus nobilis* (Haddon & Shackleton, 1894)**
10g	Polyp with more than 160 tentacles from Mediterranean Sea	***Pachycerianthus dohrni* van Beneden, 1924**
11a	Polyp with up to 60 marginal tentacles and directive labial tentacle absent	***Ceriantheopsis lineata* Stampar, Scarabino, Pastorino & Morandini, 2015**
11b	Polyp with up to 70 marginal tentacles and cnido-glandular tract at fertile mesenteries present	***Ceriantheopsis austroafricana* Molodtsova, Griffiths & Acuña, 2011**
11c	Polyp with up to 70 marginal tentacles and cnido-glandular tract at fertile mesenteries absent	***Ceriantheopsis nikitai* Molodtsova, 2001**
11d	Polyp with more than 90 marginal tentacles and short directive mesenteries	***Ceriantheopsis americana* (Agassiz in Verrill, 1864)**
12a	Polyp from India (shallow waters) with more than 150 marginal tentacles	*Cerianthus andamanensis* Alcock, 1893*
12b	Polyp from India (deep sea ~ 5000 m) with up to 40 marginal tentacles and directive labial tentacle absent	*Cerianthus valdiviae* Carlgren, 1912*
12c	Polyp from North Atlantic (deep sea ~ 5000 m) with up to 30 marginal tentacles	*Cerianthus bathymetricus* Moseley, 1877*
12d	Polyp from Red Sea (shallow waters) with up to 20 marginal tentacles	*Cerianthus medusula* (Klunzinger, 1877)*
12e	Description with information about mesentery organization and tentacle distribution	**13**
13a	Species from Pacific Ocean	**14**
13b	Species from Atlantic Ocean	**15**
14a	Protomesenteries 2 (P2) short, sterile and metamesenteries 1 (M1) reach or almost reach the aboral pore, marginal/ labial tentacles in 4 pseudocycles	***Cerianthus (?) mortenseni* Carlgren, 1924**
14b	Polyp from Japan, Korea or China, marginal tentacles in 4 pseudocycles and directive in position 2, labial tentacles in 4 pseudocycles and directive in position 3	***Cerianthus filiformis* Carlgren, 1924**
14c	Polyp from Japan, marginal tentacles in 3 pseudocycles and directive in position 2, labial tentacles in 4 pseudocycles and directive in position 2	***Cerianthus japonicus* Carlgren, 1924**
14d	Polyp from Japan, marginal tentacles in 4 pseudocycles and directive in position 2, labial tentacles in 4 pseudocycles and directive in position 2	***Cerianthus punctatus* Uchida, 1979**
14e	Polyp from Indonesia, marginal tentacles in 4 pseudocycles and directive in position 2, labial tentacles in 4 pseudocycles and directive in position 2	***Cerianthus sulcatus* Kwietniewski, 1898**
14f	Polyp from Indonesia, marginal tentacles in 2 pseudocycles and directive in position 1, labial tentacles in 4 pseudocycles and directive in position 2	***Cerianthus taedus* McMurrich, 1910**
15a	Polyp from North Sea/North Atlantic, directive labial tentacle absent, 4 mesenteries attached to siphonoglyph	***Cerianthus lloydii* Gosse, 1859**
15b	Polyp from Mediterranean Sea and Central Atlantic, directive labial tentacle present, 6 mesenteries attached to siphonoglyph	***Cerianthus membranaceus* (Gmelin, 1791)**
15c	Polyp from Norwegian Sea, directive labial tentacle present, 4 mesenteries attached to siphonoglyph	***Cerianthus vogti* Danielssen, 1890**
15d	Polyp from Namibia, mesenteries type M and m and P2 are almost of the same size	***Cerianthus malakhovi* Molodtsova, 2001**
16a	Directive labial tentacle present	**17**
16b	Directive labial tentacle absent	**18**
17a	Polyp from Atlantic Ocean, microbasic P-mastigophore absent in column	***Isarachnanthus nocturnus* (den Hartog, 1977)**
17b	Polyp from Atlantic Ocean, microbasic P-mastigophore present in column	***Isarachnanthus maderensis* (Johnson, 1861)**
17c	Polyp from Pacific Ocean, directive labial tentacle in position 2	***Isarachnanthus panamensis* Carlgren, 1924**
17d	Polyp from Pacific Ocean, directive labial tentacle in position 3	***Isarachnanthus bandanensis* Carlgren, 1924**
18a	Polyp with 6 mesenteries attached to actinopharynx, protomesenteries 2 (P2) long (3/4 of gastric cavity)	***Arachnanthus sarsi* Carlgren, 1912**
18b	Polyp with 4 mesenteries attached to actinopharynx, protomesenteries 2 (P2) short (1/2 of gastric cavity)	***Arachnanthus oligopodus* (Cerfontaine, 1891)**
18c	Polyp with 12 mesenteries attached to actinopharynx, protomesenteries 2 (P2) very short (1/4 of gastric cavity)	***Arachnanthus bockii* Carlgren, 1924**
18d	Polyp with 12 mesenteries attached to actinopharynx, protomesenteries 2 (P2) short (1/2 of gastric cavity)	***Arachnanthus australiae* Carlgren, 1937**
18e	Polyp with 8 mesenteries attached to actinopharynx, protomesenteries 2 (P2) long (almost to aboral pole)	***Arachnanthus lilith* Stampar & El Didi in Stampar et al. 2018**

The species *Cerianthus incertus*, *Cerianthus roulei*, *Cerianthus vas* and *Cerianthus stimpsonii* are not included in key due to absence of characters.

## Supplementary Material

XML Treatment for Ceriantheomorphe

XML Treatment for Ceriantheomorphe ambonensis

XML Treatment for Ceriantheomorphe brasiliensis

XML Treatment for Ceriantheomorphe adelita

XML Treatment for Ceriantheopsis

XML Treatment for Ceriantheopsis americana

XML Treatment for Ceriantheopsis austroafricanus

XML Treatment for Ceriantheopsis lineata

XML Treatment for Ceriantheopsis nikitai

XML Treatment for Cerianthus

XML Treatment for Cerianthus andamanensis

XML Treatment for Cerianthus bathymetricus

XML Treatment for Cerianthus filiformis

XML Treatment for Cerianthus incertus

XML Treatment for Cerianthus japonicus

XML Treatment for Cerianthus lloydii

XML Treatment for Cerianthus malakhovi

XML Treatment for Cerianthus medusula

XML Treatment for Cerianthus membranaceus

XML Treatment for Cerianthus mortenseni

XML Treatment for Cerianthus punctatus

XML Treatment for Cerianthus roulei

XML Treatment for Cerianthus stimpsonii

XML Treatment for Cerianthus sulcatus

XML Treatment for Cerianthus taedus

XML Treatment for Cerianthus valdiviae

XML Treatment for Cerianthus vas

XML Treatment for Cerianthus vogti

XML Treatment for Pachycerianthus

XML Treatment for Pachycerianthus aestuarii

XML Treatment for Pachycerianthus borealis

XML Treatment for Pachycerianthus curacaoensis

XML Treatment for Pachycerianthus delwynae

XML Treatment for Pachycerianthus dohrni

XML Treatment for Pachycerianthus fimbriatus

XML Treatment for Pachycerianthus insignis

XML Treatment for Pachycerianthus johnsoni

XML Treatment for Pachycerianthus longistriatus

XML Treatment for Pachycerianthus magnus

XML Treatment for Pachycerianthus maua

XML Treatment for Pachycerianthus monostichus

XML Treatment for Pachycerianthus multiplicatus

XML Treatment for Pachycerianthus nobilis

XML Treatment for Pachycerianthus schlenzae

XML Treatment for Pachycerianthus solitarius

XML Treatment for Botruanthus

XML Treatment for Botruanthus benedeni

XML Treatment for Botruanthus mexicanus

XML Treatment for Botrucnidifer

XML Treatment for Botrucnidifer norvegicus

XML Treatment for Botrucnidifer shtokmani

XML Treatment for Arachnanthus

XML Treatment for Arachnanthus australiae

XML Treatment for Arachnanthus bockii

XML Treatment for Arachnanthus oligopodus

XML Treatment for Arachnanthus lilith

XML Treatment for Arachnanthus sarsi

XML Treatment for Isarachnanthus

XML Treatment for Isarachnanthus bandanensis

XML Treatment for Isarachnanthus maderensis

XML Treatment for Isarachnanthus nocturnus

XML Treatment for Isarachnanthus panamensis

## References

[B1] Agassiz L (1859) On some new actinoid polyps of the coast of the United States. Proceedings of the Boston Society of natural History 7: 23–24.

[B2] Agassiz A (1863) On *Arachnactis brachiolata*, a species of floating Actinia found at Nahant, Massachusetts. Journal of the Boston Society of Natural History 7: 525–531.

[B3] Alcock A (1893) On some Actiniaria from the Indian seas. Journal of the Asiatic Society of Bengal 62: 151–153.

[B4] Andres A (1881) Prodromus neapolitanae actiniarum faunae addito generalis actiniarum bibliographiae catalogo. Mitteilungen aus der Zoologischen Station zu Neapel 2: 305–371.

[B5] Andres A (1883) Le Attinie. Coi Tipi der Salviucci, Roma, 460 pp.

[B6] Arai MN (1965) A new species of *Pachycerianthus*, with discussion of the genus and an appended glossary. Pacific Science 19: 205–218.

[B7] Arai MN (1971) *Pachycerianthus* (Ceriantharia) from British Columbia and Washington. Journal of the Fisheries Research Board of Canada 28: 1677–1680. 10.1139/f71-250

[B8] Arai MN (1972) The muscular system of *Pachycerianthus fimbriatus*. Canadian Journal of Zoology 50: 311–317. 10.1139/z72-042

[B9] Arai MN (1985) Electrical activity associated with withdrawal and feeding of *Pachycerianthus fimbriatus* (Anthozoa, Ceriantharia). Marine Behaviour and Physiology 12: 47–56. 10.1080/10236248509378632

[B10] Arai MN Karakashian S (1973) The fine structure of the mesogloea of the column of *Pachycerianthus fimbriatus* (Anthozoa). Publications of the Seto Marine Biological Laboratory 20: 719–729. 10.5134/175747

[B11] Arai MN Walder GL (1973) The feeding response of *Pachycerianthus fimbriatus* (Ceriantharia). Comparative Biochemistry and Physiology Part A: Physiology 44: 1085–1092. 10.1016/0300-9629(73)90246-6

[B12] Ates RML (1982) De Viltkokeranemoon; *Cerianthus lloydii*. Het Aquarium Maandbl voor Aquarium, Terr en Insektariumkd 52: 80–84.

[B13] Ates RML (1985) Kokeranemonen (Ceriantharia) … geen aanbeveling nodig! Het Zee-Aquarium Maandblad voor Zee-Aquarium Liefhebbers 35: 228–232.

[B14] Ates RML (1997) Bloemdieren: De Zeeanemonen En Hun Verwanten Van De Nederlandse Kust. C. Moerman, Zeeanjer, 31 pp.

[B15] Blanco FR (1987) Antozoos nuevos para el litoral ibérico, recolectados en Galicia. Boletin de la Real Sociedad Española de Historia Natural 83: 197–204.

[B16] Bolam SG Barry J Bolam T Mason C Rumney HS Thain JE Law RJ (2011) Impacts of maintenance dredged material disposal on macrobenthic structure and secondary productivity. Marine Pollution Bulletin 62: 2230–2245. 10.1016/j.marpolbul.2011.04.012 21868044

[B17] Braber L Borghouts CH (1977) Distribution and ecology of Anthozoa in the estuarine region of the rivers Rhine, Meuse and Scheldt. Hydrobiologia 52: 15–21. 10.1007/BF02658077

[B18] Brito A (1986) Descripcion de *Isarachnanthus cruzi*, una nueva specie de cerianthario (Cnidaria: Anthozoa: Ceriantharia) de las Islas Canarias. Vieraea 16: 173–181.

[B19] Brown CJ Collier JS (2007) Mapping benthic habitat in regions of gradational substrata: An automated approach utilizing geophysical, geological, and biological relationships. Estuarine, Coastal and Shelf Science 78: 203–214. 10.1016/j.ecss.2007.11.026

[B20] Cairns S Hartog C Arneson C (1986) Class Anthozoa (Corals, anemones). In: Sterrer W Schoepfer-Sterrer C (Eds) Marine Fauna and Flora of Bermuda.A Systematic Guide to the Identification of Marine Organisms. John Wiley & Sons Ltd., New York, 159–194.

[B21] Calado R (2006) Marine ornamental species from European waters: a valuable overlooked resource or a future threat for the conservation of marine ecosystems? Scientia Marina 70: 389–398. 10.3989/scimar.2006.70n3389

[B22] Carlgren O (1893) Studien über nordische Actinien. Kungliga Svenska Vetenskapsakademiens Handlingar 25: 1–148.

[B23] Carlgren O (1895) Jahresberichte für 1889, 1890, und 1891 über die Anthozoen. Archiv für Naturgeschichte 61: 235–298.

[B24] Carlgren O (1896) Jahresbericht über die Anthozoen für die Jahre 1892 und 1893. Archiv für Naturgeschichte 62: 145–180.

[B25] Carlgren O (1900) Ostafrikanische Actinien. Gesammelt von Herrn Dr. F. Stuhlmann 1888 und 1889. Mittheilungen aus dem Naturhistorischen Museum 17: 21–144.

[B26] Carlgren O (1906) Die Actinien-Larven. In: Brandt K Apstein C (Eds) Nordisches Plankton.Verlag von Lipsius & Tischer, Leipzig, 65–89.

[B27] Carlgren O (1912a) Ceriantharia. Danish Ingolf-Expedition 5: 1–79.

[B28] Carlgren O (1912b) Über Ceriantharien des Mittelmeers. Mitteilungen aus der Zoologischen Station zu Neapel 20: 356–391.

[B29] Carlgren O (1923) Ceriantharia und Zoantharia der Deutschen Tiefsee-Expedition. Deutsche Tiefsee-Expedition 1898–1899 19: 243–337.

[B30] Carlgren O (1924) Papers from Dr. Th. Mortensen’s Pacific expedition 1914-16. XVI. Ceriantharia. Videnskabelige Meddelelser fra Dansk Naturhistorisk Forening 75: 169–195.

[B31] Carlgren O (1927) Report on the Actiniaria and Ceriantharia. Transactions of the Zoological Society of London 22: 443–445. 10.1111/j.1096-3642.1927.tb00205.x

[B32] Carlgren O (1928) Ceriantharier, Zoantharier och Actiniarier. Meddelelser om Grønland 23 suppl: 253–308.

[B33] Carlgren O (1931) On some Ceriantharia. Arkiv för Zoologi 23: 1–10.

[B34] Carlgren O (1932) Die Ceriantharien, Zoantharien und Actiniarien des arktischen Gebietes. In Eine Zusammenstellung der arktischen Tierformen mit besonderer Berücksichtigung des Spitzbergen-Gebietes auf Grund der Ergebnisse der Deutschen Expedition in das Nördliche Eismeer im Jahre 1898. Gustav Fischer, Jena, 255–266.

[B35] Carlgren O (1937) Ceriantharia and Zoantharia. Scientific Reports of the Great Barrier Reef Expedition 1928–29 5(5): 177–207.

[B36] Carlgren O (1940) A contribution to the knowledge of the structure and distribution of the cnidae in the Anthozoa. Kungliga Fysiografiska Sällskapets Handlingar 51: 1–62.

[B37] Carlgren O (1942) Actiniaria Part II. Danish Ingolf-Expedition 5: 1–92.

[B38] Carlgren O (1945) Polypdyr (Coelenterata) III. Koraldyr. Danmarks Fauna Udgivet af Dansk Naturhistorisk Forening 51: 3–167.

[B39] Carlgren O (1951) The actinian fauna of the Gulf of California. Proceedings of the United States National Museum 101: 415–449. 10.5479/si.00963801.101-3282.415

[B40] Carlgren O Hedgpeth JW (1952) Actiniaria, Zoantharia and Ceriantharia from shallow water in the northwestern Gulf of Mexico. Publications of the Institute of Marine Science (University of Texas) 2: 143–172.

[B41] Carter S (1995) *Pachycerianthus* (Anthozoa: Cerianthidea), two newly described species from Port Jackson, Australia. Records of the Australian Museum 47: 1–6. 10.3853/j.0067-1975.47.1995.3

[B42] Casellato S Masiero L Sichirollo E Soresi S (2007) Hidden secrets of the Northern Adriatic: “Tegnúe”, peculiar reefs. Central European Journal of Biology 2: 122–136. 10.2478/s11535-007-0004-3

[B43] Cerfontaine P (1891a) Notes préliminaires sur l’organisation et le développement de différentes formes d’Anthozoaires. IV. Sur un nouveau cerianthe du golfe de Naples, *Cerianthus oligopodus* (n. sp.). Bulletin de l’Académie Royale des Sciences, des Lettres et des Beaux-Arts de Belgique (3^me^ série) 21: 32–39.

[B44] Cerfontaine P (1891b) Notes préliminaires sur l’organisation et le développement de différentes formes d’Anthozoaires. Deuxième communication. Bulletin de l’Académie Royale des Sciences, des Lettres et des Beaux-Arts de Belgique (3^me^ série) 22: 128–148.

[B45] Cerfontaine P (1909) Contribution à l’études des Cerianthides. Archives de Biologie 24: 653–707.

[B46] Ceriello H Lopes CS Dias GM Stampar SN (2019) A different manner to share a house: is a colonial species possible in Ceriantharia (Cnidaria; Anthozoa)? Marine Biodiversity 49(4): 2017–2020. 10.1007/s12526-019-00942-2

[B47] Child CM (1903a) Form regulation in *Cerianthus*. 1. The typical course of regeneration. Biological Bulletin 5: 239–260. 10.2307/1535783

[B48] Child CM (1903b) Form regulation in *Cerianthus*. 2. The effect of position, size and other factors upon regeneration. Biological Bulletin 6: 1–11. 10.2307/1535808

[B49] Child CM (1904a) Form regulation in *Cerianthus*. 3. The initiation of regeneration. Biological Bulletin 6: 55–74. 10.2307/1535589

[B50] Child CM (1904b) Form regulation in *Cerianthus*. 4. The role of water-pressure in regeneration. Biological Bulletin 6: 266–286. 10.2307/1535820

[B51] Child CM (1908) Form regulation in *Cerianthus æstuarii*. Biological Bulletin 15: 27–53. 10.2307/1535562

[B52] Chintiroglou C den Hartog JC (1995) Additional records of Actiniaria (Anthozoa) from Greece. Zoologische Mededelingen 69: 353–364.

[B53] Chintiroglou C Koukouras A (1991) Observations on the feeding habits of *Calliactis parasitica* (Couch, 1842), Anthozoa, Cnidaria. Oceanologica Acta 14: 389–396.

[B54] Çinar ME Yokes MB Açik S Bakir AK (2014) Checklist of Cnidaria and Ctenophora from the coasts of Turkey. Turkish Journal of Zoology 38: 677–697. 10.3906/zoo-1405-68

[B55] Coolen JWP Bos OG Glorius S Lengkeek W Cuperus J van der Weide B Agüera A (2015) Reefs, sand and reef-like sand: A comparison of the benthic biodiversity of habitats in the Dutch Borkum Reef Grounds. Journal of Sea Research 103: 84–92. 10.1016/j.seares.2015.06.010

[B56] Cosentino A Potoschi S Giacobbe A (2011) The presence of *Phoronis australis* (Phoronida) in southern Italian waters. Biogeographia 30: 409–415. 10.21426/B630110602

[B57] Cutress CE (1961) *Habrosanthus bathamae*, n. gen., n. sp. (Actiniaria: Sagartiidae) from New Zealand. Transactions of the Royal Society of New Zealand 1: 95–101.

[B58] Cutress CE (1977) Corallimorpharia, Actiniaria, Ceriantharia. In: Devaney DM Eldredge LG (Eds) Reef and Shore Fauna of Hawaii.Bishop Museum Press, Honolulu, 130–147.

[B59] Cutress CE Arneson CA (1987) Sea anemones of Enewetak Atoll. In: Devaney DM Reese ES Burch BL Helfrich P (Eds) The Natural History of Enewetak Atoll.Office of Scientific and Technical Information, US Department of Energy, Honolulu, 53–62.

[B60] Daly M Brugler MR Cartwright P Collins AG Dawson MN Fautin DG France SC McFadden CS Opresko DM Rodriguez E Romano SL Stake JL (2007) The phylum Cnidaria: A review of phylogenetic patterns and diversity 300 years after Linnaeus. Zootaxa, 127–182. 10.11646/zootaxa.1668.1.11

[B61] Danielssen DC (1860) Forhandlinger i Videnskabs-Selskabet i Christiania On Cerianthus borealis n. sp. Trykt hos Brøgger & Christie, Christiania, 251 pp.

[B62] Danielssen DC (1888) *Cerianthus borealis*. Bergens Museums Aarsberetning 1: 1–12.

[B63] Danielssen DC (1890) Actinida. In: Den Norske Nordhavs-Expedition 1876–1878. Zoologi. Grøndahl and Søn, Christiania, 184.

[B64] de Blainville HMD (1830) Dictionnaire des Sciences Naturelles. FG Levrault, Paris, 631 pp.

[B65] de Blainville HMD (1834) Manuel d’Actinologie ou de Zoophytologie. FG Levrault, Paris, 644 pp 10.5962/bhl.title.8768

[B66] Delle Chiaje S (1841) Descrizione e anotomia degli animali invertebrati della Sicilia Citeriore osservati vivi negli anni 1822–1830. C. Batelli e Comp., Napoli, 420 pp 10.5962/bhl.title.10031

[B67] den Hartog JC (1977) Descriptions of two new Ceriantharia from the Caribbean region, *Pachycerianthus curancaoensis* n. sp. and *Arachnanthus nocturnus* n. sp. with a discussion of the Cnidom and of the classification of the Ceriantharia. Zoologische Mededelingen 51: 211–242.

[B68] den Hartog JC (1997) The sea anemone fauna of Indonesian coral reefs. In: Tomascik T Mah AJ Nontji A Moosa MK (Eds) The ecology of the Indonesian seas 1.Periplus Editions, Singapore, 351–370.

[B69] Dons C (1945) Norges strandfauna XXXII hexakoraller. Kongelige Norske Videnskabers Selskab Forhandlinger 18: 17–20.

[B70] Duerden JE (1902a) Report on the actinians of Porto Rico. Bulletin of the U.S. Fisheries Commission 20: 323–374.

[B71] Duerden JE (1902b) On the actinian *Bunodeopsis globulifera*, Verrill. Transactions of the Linnean Society 8: 297–317. 10.1111/j.1096-3642.1902.tb00478.x

[B72] Eleftheriou A Basford DJ (1983) The general behaviour and feeding of *Cerianthus lloydii* Gosse. Cahiers de Biologie Marine 24: 147–158.

[B73] Emig CC Herberts C Thomassin BA (1972) Sur l’association de *Phoronis australis* avec *Cerianthus maua* dans les zones récifales de Madagascar. Marine Biology 15: 304–315. 10.1007/BF00401390

[B74] Faurot L (1895) Études sur l’anatomie, l’histologie et le développement des actinies. Archives de Zoologie Expérimentale et Générale 3: 43–262.

[B75] Fautin DG (1998) Class Anthozoa: Orders Actiniaria, Ceriantharia, and Zoantharia. In: Scott PV Blake JV (Eds) , Taxonomic Atlas of the Benthic Fauna of the Santa Maria Basin and Western Santa Barbara Channel.Santa Barbara Museum of Natural History, Santa Barbara, 113–139.

[B76] Fautin DG (2013) Hexacorallians of the World. http://geoportal.kgs.ku.edu/hexacoral/anemone2/index.cfm

[B77] Fautin DG Hickman Jr. CP Daly M Molodtsova TN (2007) Shallow-Water Sea Anemones (Cnidaria: Anthozoa: Actiniaria) and Tube Anemones (Cnidaria: Anthozoa: Ceriantharia) of the Galápagos Islands. Pacific Science 61: 549–573. 10.2984/1534-6188(2007)61[549:SSACAA]2.0.CO;2

[B78] Field LR (1949) Sea Anemones and Corals of Beaufort, North Carolina. Duke University Press, Durham, 39 pp.

[B79] Fischer P (1874) Recherches sur les actinies des cotes océaniques de France. Nouvelles Archives du Muséum d’Histoire Naturelle de Paris 10: 193–244.

[B80] Fischer P (1875) Anthozoaires du département de la Gironde et des côtes du sud-ouest de la France. Actes de la Société Linnéenne de Bordeaux 30: 183–192.

[B81] Fischer P (1887) Contribution a l’actinologie française. Archives de Zoologie Expérimentale et Générale 5: 381–442.

[B82] Fischer P (1889) Nouvelle contribution à l’actinologie française. Première Partie. Actinies d’Arcachon (Gironde). Actes de la Société Linnéenne de Bordeaux 43: 252–309.

[B83] Fishelson L (1970) Littoral fauna of the Red Sea: the population of non-scleractinian anthozoans of shallow waters of the Red Sea (Eilat). Marine Biology 6: 106–116. 10.1007/BF00347239

[B84] Forbes E (1843) Retrospective comments. Annals and Magazine of Natural History 12: 40–42. 10.1080/03745484309442482

[B85] Mejia ACF Molodtsova T Östman C Bavestrello G Rouse GW (2019) Molecular phylogeny of Ceriantharia (Cnidaria: Anthozoa) reveals non-monophyly of traditionally accepted families. Zoological Journal of the Linnean Society 10.1093/zoolinnean/zlz158

[B86] Fowler GH (1897) Contributions to our knowledge of the plankton of the Faroe Channel. III. The latter development of *Arachnactis albida* (M. Sars) with notes on *Arachnactis bournei* (sp.n.). Proceedings of the Zoological Society of London 65: 803–808. 10.1111/j.1096-3642.1897.tb03123.x

[B87] Frey RW (1970) The Lebensspuren of some common marine invertebrates near Beaufort, North Carolina. II. Anemone burrows. Journal of Paleontology 44: 308–211.

[B88] Füller H (1957) Exkursionsfauna von Deutschland. Wirbellose I. Volk und Wissen Volkseigener, Berlin, 485 pp.

[B89] Gili J-M (1982) Fauna de Cnidaris de les Illes Medes. KETRES, Barcelona, 175 pp.

[B90] Gmelin JF (1791) Caroli a Linné. Systema naturae per regna tria naturae: secundum classes, ordines, genera, species, cum characteribus, differentiis, synonymis, locis. 1 (6). Beer, Leipzig, 3021–3909.

[B91] Goette A (1897) Einiges über die Entwickelung der Scyphopolypen. Zeitschrift für Wissenschaftliche Zoologie 63: 292–378.

[B92] González-Muñoz R Simões N Guerra-Castro EJ Hernández-Ortíz C Carrasquel G Mendez E Lira C Rada M Hernández I Pauls SM Croquer A Cruz-Motta JJ (2016) Sea anemones (Cnidaria: Actiniaria, Corallimorpharia, Ceriantharia, Zoanthidea) from marine shallow-water environments in Venezuela: new records and an updated inventory. Marine Biodiversity Records 9: 1–35. 10.1186/s41200-016-0016-7

[B93] Gosse PH (1856a) *Edwardsia vestita* (Forbes). Annals and Magazine of Natural History, Series 2, 18: 73–74. 10.1080/00222935608697585

[B94] Gosse PH (1856b) On *Edwardsia carnea*, a new British Zoophyte. Annals and Magazine of Natural History, Series 2, 18: 219–211. 10.1080/00222935608697624

[B95] Gosse PH (1858) Synopsis of the families, genera, and species of the British Actiniae. Annals and Magazine of Natural History, Series 3, 1: 414–419. 10.1080/00222935808696950

[B96] Gosse PH (1859) Characters and descriptions of some new British sea-anemones. Annals and Magazine of Natural History, Series 3, 3: 46–50.

[B97] Gosse PH (1860) A History of the British Sea-Anemones and Corals. Van Voorts, London, 362 pp 10.5962/bhl.title.27151

[B98] Goy J (1979) Campagne de la Calypso au large des côtes atlantiques de l’Amérique du Sud (1961–1962); 35. Méduses. Résultats scientifiques des campagnes de la Calypso 11: 263–296.

[B99] Graeffe E (1884) Seethierfauna des Golfes von Triest. Arbeiten aus dem Zoologischen Instituten Universität Wien 5: 333–362.

[B100] Gravier C (1902) Sur un cérianthaire pélagique adulte. Comptes Rendus Hebdomadaires des Séances de l’Académie des Sciences 135: 591–593.

[B101] Gravier C (1904) Recherches sur un cérianthaire pélagique du Golfe de Californie (*Dactilactis benedeni*, n. sp.). Annales des Sciences Naturelles 20: 253–294.

[B102] Gravier C (1922) Hexactinidés provenant des campagnes des yachts Hirondelle I et II et Princesse-Alice I et II (1888–1913). In: Résultats des Campagnes Scientifiques Accomplies sur son Yacht par Albert I^er^ Prince Souverain de Monaco Publiés sous sa Direction avec le Concours de M. Jules Richard. Imprimerie de Monaco, Monaco, 3–104.

[B103] Gray JE (1867) Notes on Zoanthinæ, with the descriptions of some new genera. Proceedings of the Zoological Society of London 15: 233–240.

[B104] Grebel’nyi SD (2001) Hexacorallia. Explorations of the Fauna of the Seas 51: 36–38.

[B105] Grieg JA (1913) Bidrag til knudskapen om Hardangerfjordens fauna. Bergens Museums Aarbok 1: 3–147.

[B106] Grube AE (1840) Actinien, Echinodermen und Würmer des Adriatischen und Mittelmeers [nach eigenen Sammlungen beschrieben von Dr. Adolph Eduard Grube]. J. H. Bon, Königsberg, 92 pp.

[B107] Haddon AC (1898) The Actiniaria of Torres Straits. Scientific Transactions of the Royal Dublin Society 6: 393–520.

[B108] Haddon AC Shackleton AM (1893) Description of some new species of Actiniaria from Torres Straits. Scientific Proceedings of the Royal Dublin Society 8: 116–131.

[B109] Haime J (1854) Memoire sur le cérianthe (*Cerianthus membranaceus*). Annales des Sciences Naturelles 1: 341–389.

[B110] Haldar BP (1981) On the climatology of the Beyt Island, western India, with special reference to the systematics of *Phoronis australis* Haswell (Phoronida). Indian Journal of Zootomy 22: 59–63.

[B111] Haque MM (1977) Some littoral coelenterates of Bangladesh and Pakistan coasts. Bangladesh Journal of Zoology 1: 33–40.

[B112] Harger O Smith SI (1876) Report on the dredgings in the region of St. George’s Banks, in 1872. Transactions of the Connecticut Academy of Arts and Sciences 3: 1–57.

[B113] Hargitt CW (1912) The Anthozoa of the Woods Hole region. Bulletin of the Bureau of Fisheries 32: 221–254.

[B114] Harms J (1993) Check list of species (algae, invertebrates and vertebrates) found in the vicinity of the island of Helgoland (North Sea, German Bight); a review of recent records. Helgoländer Meeresuntersuchungen 47: 1–34. 10.1007/BF02366182

[B115] Haurtlaub C (1884) Die Coelenteraten Helgolands. Vorläufiger Bericht. Wissenschaftliche Meeresuntersuchungen 1: 161–206.

[B116] Hedgpeth JW (1954) Anthozoa: the anemones. Fisheries Bulletin of the Fish and Wildlife Service (US) 55: 285–290.

[B117] Heider AV (1879) *Cerianthus membranaceus* Haime. Ein Beitrag zur Anatomie der Actinien. Sitzungsberichte der Kaiserlichen Akademie der Wissenschaften in Wien, Mathematisch-Naturwissenschaftliche 79: 204–254.

[B118] Heller C (1868) Die Zoophyten und Echinodermen des adriatischen Meeres. Druck von Carl Ueberreuter, Wien, 86 pp 10.5962/bhl.title.11393

[B119] Hertwig R (1882) Die Actinien der Challenger Expedition. Gustav Fischer, Jena, 119 pp.

[B120] Hertwig R (1888) Report on the Actiniaria dredged by HMS Challenger during the years 1873–1876 [Supplement]. Report on the Scientific Results of the Voyage of the H.M.S. Challenger during the years 1873–76 (Zoology) 26: 1–56. 10.5962/bhl.title.11290

[B121] Hickson SJ (1889) Coelenterata. Zoological Record 26: 1–24.

[B122] Holohan BA Klos EG Oviatt CA (1998) Population density, prey selection, and predator avoidance of the burrowing anemone (*Ceriantheopsis americanus*) in Narragansett Bay, Rhode Island. Estuaries and Coasts 21: 466–469. 10.2307/1352844

[B123] Ilmoni I (1830) Dr. Ilmoni aus Finnland schickte folgende Beiträge zur Naturgeschichte der Actinien ein. Isis von Oken 23: 694–699.

[B124] Ilmoni I (1831) Sur deux espèces nouvelles de la famille des actinies. Bulletin des Sciences Naturelles 24: 123.

[B125] Jensen P (1992) *Cerianthus vogti* Danielssen, 1890 (Anthozoa: Ceriantharia). A species inhabiting an extended tube system deeply buried in deep-sea sediments off Norway. Sarsia 77: 75–80. 10.1080/00364827.1992.10413494

[B126] Johnson JY (1861) Notes on the sea-anemones of Madeira, with descriptions of new species. Proceedings of the Zoological Society of London 1861: 298–306.

[B127] Jonsson LG Lundälv T Johannesson K (2001) Symbiotic associations between anthozoans and crustaceans in a temperate coastal area. Marine Ecology Progress Series 209: 189–195. 10.3354/meps209189

[B128] Jourdan E (1880) Recherches zoologiques et histologiques sur les zoanthaires du Golfe de Marseille. Annales des Sciences Naturelles 10: 1–154. 10.5962/bhl.title.4956

[B129] Kayal E Roure B Philippe H Collins AG Lavrov DV. (2013) Cnidarian phylogenetic relationships as revealed by mitogenomics. BMC Evolutionary Biology 13: 5. 10.1186/1471-2148-13-5 PMC3598815 23302374

[B130] Keegan BF Könnecker G (1973) In situ quantitative sampling of benthic organisms. Helgoländer Wissenschaftliche Meeresuntersuchungen 24(1–4): 256–263. 10.1007/BF01609516

[B131] Kelly E Keegan BF (2000) Case 3111. *Pachycerianthus* Roule, 1904 (Cnidaria, Anthozoa): Proposed Designation of *P. multiplicatus* Carlgren, 1912 as the Type Species. Bulletin of Zoological Nomenclature 57: 11–13. 10.5962/bhl.part.20659

[B132] Kingsley JS (1904) A description of *Cerianthus borealis* Verrill. Tufts College Studies 1: 345–361.

[B133] Kiseleva MI (1975) Food spectra of some benthic invertebrates in the Black Sea. Zoologischeskii Zhurnal1 54: 1596–1601.

[B134] Klunzinger CB (1877) Die Korallthiere des Rothen Meeres. 1: Die Alcyonarien und Malacodermen, 1^st^ ed. Gutmann’schen Buchhandlung, Berlin, 98 pp, 8 pls.

[B135] Koren J Danielssen DC (1877) Beskrivelse over nogle nye norske Coelenterater [Description of some new Norwegian coelenterates]. In: Koren J Danielssen DC (Eds) Fauna Littoralis Norvegiae.JD Beyer, Bergen, 77–81.

[B136] Krempf A (1905) Liste des Hexanthides rapportés de l’Océan Indien (Golfe de Tadjourah) par M. Ch. Gravier. Bulletin du Muséum national d’Histoire naturelle 11: 191–196.

[B137] Kristensen E Aller RC Aller JY (1991) Oxic and anoxic decomposition of tubes from the burrowing sea anemone *Ceriantheopsis americanus*: Implications for bulk sediment carbon and nitrogen balance. Journal of Marine Research 49: 589–617. 10.1357/002224091784995774

[B138] Kussakin G Kostina EE (1996) The Intertidal Biota of Volcanic Yankich Island (Middle Kuril Islands). Publications of the Seto Marine Biological Laboratory 37: 201–225. 10.5134/176267

[B139] Kwietniewski CR (1898) Actiniaria von Ambon und Thursday Island. In: Zoologische Forschungsreisen in Australien und dem Malayischen Archipel von Richard Semon. Gustav Fischer, Jena, 385–430.

[B140] Laverack MS Blackler M (1974) Fauna and Flora of St. Andrews Bay. Scottish Academic Press, Edinburgh, 310 pp.

[B141] Leloup E (1931) Contribution a la repartition des cerianthaires dans le sud de la Mer du Nord. Bulletin du Musée Royal d’Histoire Naturelle de Belgique 7: 1–10.

[B142] Leloup E (1932) Cerianthaires de l´Ocean Atlantique. Bulletin du Musée Royal d’Histoire Naturelle de Belgique 8: 1–19.

[B143] Leloup E (1960) Larves de Cérianthaires de Monaco et de Villefranche-sur-Mer. Bulletin de l’Institut Océanographique 1185: 1–19.

[B144] Leloup E (1962) AnthozoaCeriantharia: larvae. Conseil International pour l’Exploration de la mer 93: 1–7.

[B145] Leloup E (1964) Larves de cerianthaires. Discovery Reports 33: 251–307.

[B146] Levinsen GMR (1893) Annulata, Hydroidae, Anthozoa, Porifera. In: Petersen CGJ (Ed.) , Videnskabelige Udbytte af Kanonbaaden “Hauchs” togter i de Danske Have indenfor Skagen i Aarene 1883–1886.Host, Copenhagen, 307–464.

[B147] Lo Bianco S (1909) Notizie biologiche riguardanti specialmente il periodo di maturità sessuale degli animali del golfo di Napoli. Mitteilungen aus der Zoologischen Station zu Neapel 19: 513–716.

[B148] Lo Iacono C Orejas C Gori A Gili JM Requena S Puig P Ribó M (2012) Habitats of the Cap de Creus continental shelf and Cap de Creus canyon, northwestern Mediterranean. In: Harris PT Baker EK (Eds) , Seafloor Geomorphology as Benthic Habitat-GeoHAB Atlas of Seafloor Geomorphic Features and Benthic Habitats.Elsevier, Amsterdam, 457–469. 10.1016/B978-0-12-385140-6.00032-3

[B149] Lopes CSS Ceriello H Morandini AC Stampar SN (2019) Revision of the Genus *Ceriantheomorphe* (Cnidaria, Anthozoa, Ceriantharia) with description of a new species from the Gulf of Mexico and northwestern Atlantic. ZooKeys 874: 127–148. 10.3897/zookeys.874.35835 31565021 PMC6746745

[B150] Lütken C (1861) Nogle Bémærkninger om de ved de danske Kyster iagttagne Arter af Aktiniernes Gruppe. Videnskabelige Meddelelser fra Dansk Naturhistorisk Forening 14: 184–200.

[B151] Lütken C (1889) Nogle temmelig uventede Forøgelser af den norske Havfauna. Videnskabelige meddelelser fra den Naturhistoriske forening i Kjöbenhavn 7: 358–362.

[B152] MacGinitie GE (1955) Distribution and ecology of the marine invertebrates of Point Barrow, Alaska. Smithsonian Miscellaneous Collections 128: 1–120.

[B153] Manuel RL (1977) A redescription of *Edwardsia beautempsi* and *E. timida* (Actiniaria: Edwardsidae). Cahiers de Biologie Marine 18: 483–497.

[B154] Manuel RL (1981) British Anthozoa keys and notes for the identification of the species. Academic Press, London, 241 pp.

[B155] Mariscal RN Conklin EJ Bigger CH (1977) The Ptychocyst, a major new category of cnida used in tube construction by a cerianthid anemone. Biological Bulletin 152: 392–405. 10.2307/1540427

[B156] Mark EL (1884) Selections from embryological monographs. III. Polyps. Memoirs of the Museum of Comparative Zoology at Harvard College 9: 5–52.

[B157] Mata SA Corsetti CL Corsetti FA Awramik SM Bottjer DJ (2012) Lower Cambrian anemone burrows from the upper member of the Wood Canyon Formation, Death Valley region, United States. Palaios 27: 594–606. 10.2110/palo.2012.p12-016r

[B158] McFarlane ID (1988) Variability in the startle response of *Pachycerianthus multiplicatus* (Anthozoa: Ceriantharia). Comparative Biochemistry and Physiology Part A: Physiology 89: 365–370. 10.1016/0300-9629(88)91041-9

[B159] McMurrich JP (1887) Notes on the fauna of Beaufort, North Carolina. Studies at the Biological Laboratory of the Johns Hopkins University 4: 55–63.

[B160] McMurrich JP (1893) Report on the Actiniæ collected by the United States Fish Commission Steamer Albatross during the winter of 1887–1888. Proceedings of the United States National Museum 16: 119–216. 10.5479/si.00963801.16-930.119

[B161] McMurrich JP (1910) Actiniaria of the Siboga expedition, Part I. Ceriantharia. Siboga-Expeditie Monographes 10: 1–48.

[B162] Mello-Leitão CF (1919) *Cerianthus Brasiliensis*; um novo cerianthoide americano. Archivos da Escola Superior de Agricultura e Medicina Veterinaria 3: 35–39.

[B163] Menon KR (1927) Subclass Zoantharia (except Scleractiniae). Bulletin of Madras Government Museum 1: 31–40.

[B164] Milne Edwards H (1857) Histoire Naturelle des Coralliaires ou Polypes Proprement Dits, vol. 1, 1^st^ ed. Librairie Encyclopédique de Roret, Paris, 326 pp 10.5962/bhl.title.11574

[B165] Milne Edwards H Haime J (1851) Archives du Muséum d’Histoire Naturelle. 5 Monographie des Polypiers fossiles des terrains paléozoïques, précédée d’un tableau général de la classification des Polypes. Gide et J Baudry, Paris, 502 pp.

[B166] Miranda LS Marques AC (2016) Hidden impacts of the Samarco mining waste dam collapse to Brazilian marine fauna-an example from the staurozoans (Cnidaria). Biota Neotropica 16 (2): e20160169 10.1590/1676-0611-BN-2016-0169

[B167] M’Intosh WC (1875) The Marine Invertebrates and Fishes of St. Andrews. Taylor and Francis, London, 186 pp 10.5962/bhl.title.6247

[B168] Molodtsova TN (2000) Fauna ceriantariy atlanticheskogo okeana I sostav roda Cerianthus mirovoy fauny. Aftoreferat na soiskanie uchenoy stepeni kandidata biologicheskih nauk. Dialog-MGU, Moscow, 21 pp.

[B169] Molodtsova TN (2001a) Cerianthids (Anthozoa, Cnidaria) of the region of Bengual upwelling 1. *Ceriantheopsis nikitai* n.sp. Zoologicheskii Zhurnal 80: 773–780. [original in Russian]

[B170] Molodtsova TN (2001b) Cerianthids (Anthozoa, Cnidaria) of the region of Bengual upwelling 2. *Cerianthus malakhovi* n. sp. and composition of the genus Cerianthus. Zoologicheskii Zhurnal 80: 909–920. [original in Russian]

[B171] Molodtsova TN (2001c) Cerianthids (Anthozoa, Cnidaria) from Bengual upwelling region. 3. *Botrucnidifer shtockmani*. Zoologicheskii Zhurnal 80: 1027–1037.

[B172] Molodtsova TN (2001d) On the taxonomic status of *Cerianthus septentrionalis* van Beneden, 1923 (Cnidaria: Anthozoa: Ceriantharia). Zoosystematica Rossica 10: 9–10.

[B173] Molodtsova TN (2003) On *Isarachnanthus* from Central Atlantic and Caribbean region with notes on *Isarachnactis lobiancoi* (Carlgren, 1912). Zoologische Verhandelingen 345: 249–255.

[B174] Molodtsova TN (2004a) Ceriantharia (Cnidaria: Anthozoa) from the Faroe Islands. Fróðskaparrit 51: 292–297.

[B175] Molodtsova TN (2004b) On the taxonomy and presumable evolutionary pathways of planktonic larvae of Ceriantharia (Anthozoa, Cnidaria). Hydrobiologia 530–531: 261–266. 10.1007/s10750-004-2671-7

[B176] Molodtsova TN (2007) Tube anemones (CerianthariaAnthozoa) of New Caledonia. Compendium of marine species of New Caledonia. Documents scientifiques et techniques, IRD, II7: 133.

[B177] Molodtsova TN (2009) Ceriantharia (Cnidaria) of the Gulf of Mexico. In: Gulf of Mexico Origin, Waters, and Biota: Volume I, Biodiversity. Texas A&M University Press, College Station, 365–367.

[B178] Molodtsova TN (2014) Deep-sea fauna of European seas: An annotated species check-list of benthic invertebrates living deeper than 2000 m in the seas bordering Europe. Ceriantharia. Invertebrate Zoology 11(1): 99–100. 10.15298/invertzool.11.1.09

[B179] Molodtsova TN Malakhov VV (1995a) *Cerianthus lloydii* (Anthozoa, Ceriantharia) from the volcanic ecosystem of Kraternaya Bay. 1. Morphology and anatomy of adult polyps, geographic distribution. Zoologicheskii Zhurnal 74(10): 5–17.

[B180] Molodtsova TN Malakhov VV (1995b) *Cerianthus lloydii* (Anthozoa, Ceriantharia) from a volcanic ecosystem of Kraternaya Bay. II. Larval development. Zoologicheskii Zhurnal 74(11): 4–11.

[B181] Molodtsova TN Griffiths CLCF Acuña FH (2011) A new species of shallow-water cerianthid (Cnidaria: Anthozoa) from South Africa, with remarks on the genus *Ceriantheopsis*. African Natural History 7: 1–8.

[B182] Molodtsova T (2020) World List of Ceriantharia Ceriantharia Accessed through: World Register of Marine Species at: https://www.marinespecies.org/aphia.php?p=taxdetails&id=1361 on 2020-04-16

[B183] Moore PG Cameron KS (1999) A note on a hitherto unreported association between *Photis longicaudata* (Crustacea: Amphipoda) and *Cerianthus lloydii* (Anthozoa: Hexacorallia). Journal of the Marine Biological Association of the United Kingdom 79: 369–370. 10.1017/S0025315498000447

[B184] Morri C Bavestrello G Bianchi CN (1991) Faunal and ecological notes on some benthic cnidarian species from the Tuscan Archipelago and eastern Ligurian sea (Western Mediterranian). Bollettino dei musei e degli istituti biologici dell’Università di Genova 54/55: 27–47.

[B185] Moseley HN (1877) On new forms of Actiniaria dredged in the deep sea; with a description of certain pelagic surface-swimming species. Transactions of the Linnean Society 1: 295–305. 10.1111/j.1096-3642.1877.tb00444.x

[B186] Müllegger S (1938) Die Aktinien: Beschreibung der vornehmlich im Aquarium gehaltenen Aktinien. Wochenschrift für Aquarien und Terrarienkunde 39–44: 1–16.

[B187] Nair RV (1949) On two new ceriantharian larvae from the Madras plankton. Records of the Indian Museum 47: 239–251.

[B188] Nakamoto D (1919) A new species of *Cerianthus*. Zoological Magazine (Tokyo) 31: 118–120.

[B189] Nakamoto D (1923) *Cerianthus misakiensis* n. sp. Zoological Magazine (Tokyo) 35: 167–172.

[B190] Naumov D V (1968) Coelenterata. In: Opredelitel fauny Chernogo I Azovskogo. Naukova Dumka, Kiev, 56–74.

[B191] Nienhaus K Renzi F Vallone B Wiedenmann J Nienhaus GU (2006) Exploring chromophore − protein interactions in fluorescent protein cmFP512 from Cerianthus membranaceus: X-ray structure analysis and optical spectroscopy. Biochemistry 45(43): 12942–12953. 10.1021/bi060885c 17059211

[B192] Nyholm K-G (1943) Zur Entwicklung und Entwicklungsbiologie der Ceriantharien und Aktinien. Zoologiska Bidrag från Uppsala 22: 87–248.

[B193] Ocaña MA Tocino LS López González S Viciana Martín JF (2000a) Guía submarina de invertebrados no artrópodos. Albolote, Granada, 417 pp.

[B194] Ocaña O Tocino LS Lopéz-González PJ (2000b) Consideraciones faunística y biogeográficas de los antozoos (Cnidaria: Anthozoa) de la costa de Granada (Mar de Alborán). Zoologica Baetica 11: 51–65.

[B195] Panikkar NK (1936) On *Apiactis bengalensis*, species nova, a new pelagic larval ceriantharian from the Madras plankton. Zoologischer Anzeiger 140: 250–260.

[B196] Panikkar NK (1947) Observations on the structure and developmental stages of a new species of *Arachnactis* from the Madras plankton. Annales des Sciences Naturelles 9: 227–250.

[B197] Parker GH (1900) Synopses of North American invertebrates. XIII. The Actiniaria. American Naturalist 34: 747–758. 10.1086/277763

[B198] Pax F (1908) Anthozoa. Die Aktinienfauna Westafrikas. Denkschriften der Medizinisch-Naturwissenschaftlichen Gesellschaft 13: 261–302.

[B199] Pax F (1909) Die Aktinien der ostafrikanischen Inseln. In: Voeltzkow A (Ed.) , Reise in Ostafrika in den Jahren 1903–1905.E Schweizerbart Verlagsbuchhandlung, Stuttgart, 399–418.

[B200] Pax F (1910) Studien an westindischen Actinien. Zoologische jahrbucher. Abteilung fur allgemeine zoologie und physiologie der tiere suppl 11: 157–330.

[B201] Pax F (1924) Actiniarien, Zoantharien und Ceriantharien von Curaçao. Kungliga Zoologisch Genootschap Natura Artis Magistra 23: 93–122.

[B202] Pax F (1928) Anthozoa. In: Dahl F (Ed.) , Die Tierwelt Deutschlands und der angrenzenden Meeresteile nach ihren Merkmalen und nach ihrer Lebensweise.Gustav Fischer, Jena, 190–237.

[B203] Pax F Müller I (1955) Gli Antozoi del Museo Civico di Storia Naturale di Trieste Parte I: Antipatharia, Ceriantharia, Zoantharia, Actiniaria, Alcyonaria e Pennatularia. Atti del Museo Civico di Storia Naturale Trieste 20: 103–129.

[B204] Pax F Müller I (1962) Fauna antozoa Jadrana. Fauna et Flora Adriatica 3: 1–343.

[B205] Peckett FJ Glegg GA Rodwell LD (2014) Assessing the quality of data required to identify effective marine protected areas. Marine Policy 45: 333–341. 10.1016/j.marpol.2013.09.013

[B206] Pei Z (1998) CoelenterataActiniariaCerianthariaZoanthidea. Science Press, Beijing, 286 pp.

[B207] Perrier E (1893) Traité de Zoologie. Librairie S. Savy, Paris 1(2): 1–864.

[B208] Peteya DJ (1973a) A light and electron microscope study of the nervous system of *Ceriantheopsis americanus* (Cnidaria, Ceriantharia). Zeitschrift für Zellforschung und Mikroskopische Anatomie 141: 301–317. 10.1007/BF00307408 4147590

[B209] Peteya DJ (1973b) A possible proprioceptor in *Ceriantheopsis americanus* (Cnidaria, Ceriantharia). Zeitschrift für Zellforschung und mikroskopische Anatomie 144: 1–10. 10.1007/BF00306682 4149114

[B210] Picton BE (1985) Anthozoans (Coelenterata: Anthozoa) new to Ireland and new records of some rarely recorded species. Irish Naturalists Journal 21: 484–488.

[B211] Picton BE Manuel RL (1985) *Arachnanthus sarsi* Carlgren, 1912: a redescription of a cerianthid anemone new to the British Isles. Zoological Journal of the Linnean Society 83: 343–349. 10.1111/j.1096-3642.1985.tb01180.x

[B212] Pirtle JL Ibarra SN Eckert GL (2012) Nearshore subtidal community structure compared between inner coast and outer coast sites in Southeast Alaska. Polar Biology 35: 1889–1910. 10.1007/s00300-012-1231-2

[B213] Rapp W (1829a) Ueber den Bau einiger Polypen des mittelländichen Meeres. Nova Acta Academiae Caesareae Leopoldino-Coralinae Naturae Curiosorum 14: 653–658.

[B214] Rapp W (1829b) Ueber die Polypen im Allgemeinen und die Actinien. Grolsherzogl. Sdch, Weimar, 62 pp.

[B215] Rastorgueff PA Bellan-Santini D Bianchi CN Bussotti S Chevaldonn P Guidetti P Harmelin JG Montefalcone M Morri C Perez T Ruitton S Vacelet J Personnic S (2015) An ecosystem-based approach to evaluate the ecological quality of Mediterranean undersea caves. Ecological Indicators 54: 137–152. 10.1016/j.ecolind.2015.02.014

[B216] Reft AJ Daly M (2012) Morphology, distribution, and evolution of apical structure of nematocysts in Hexacorallia. Journal of morphology 273: 121–136. 10.1002/jmor.11014 21960117

[B217] Rehm P Rachor E (2007) Benthic macrofauna communities of the submersed Pleistocene Elbe valley in the southern North Sea. Helgoland Marine Research 61: 127–134. 10.1007/s10152-007-0060-0

[B218] Reiner SA (1807) Prospetto della Classe dei Vermi. In: Prodromus osserv. Venezia 1804–1807. Unavailable publication, ICZN Opinion 316, Padua, 15–27.

[B219] Riemann-Zürneck K (1969) *Sagartia troglodytes* (Anthozoa). Biologie und Morphologie einer schlickbewohnenden Aktinie. Veröffentlichungen des Institutes für Meeresforschung Bremerhaven 12: 169–230.

[B220] Rioja Y Martin J (1906) Datos para el conocimiento de la fauna marina de España. Boletin de la Real Sociedad Española de Historia Natural 6: 275–281.

[B221] Ritzmann O Jokat W Czuba W Guterch A Mjelde R Nishimura Y (2004) A deep seismic transect from Hovgård Ridge to northwestern Svalbard across the continental-ocean transition: A sheared margin study. Geophysical Journal International 157(2): 683–702. 10.1111/j.1365-246X.2004.02204.x

[B222] Robertson D (1876) On the sea anemones of the shores of the Cumbraes. Proceedings of the Natural History Society of Glasgow 2: 24–30.

[B223] Robins MW (1969) The marine flora and fauna of the Isles of Scilly. Cnidaria and Ctenophora. Journal of Natural History 3: 329–343. 10.1080/00222936900770291

[B224] Rodriguez C Marques AC Stampar SN Morandini AC Christiansen E Genzano G Mianzan H (2011) The taxonomic position of the pelagic “staurozoan” *Tessera gemmaria* as a ceriantharian larva. Zootaxa 2971: 49–58. 10.11646/zootaxa.2971.1.5

[B225] Roule L (1904) Note préliminaire sur quelques formes nouvelles de cerianthaires. Comptes Rendus de l’Association Française pour l’Avancement des Sciences 32: 791–793.

[B226] Roule L (1905) Description des antipathaires et cerianthaires recueillis par S.A.S. le Prince de Monaco dans l’Atlantique nord (1886–1902). Résultats des Campagnes Scientifiques Accomplies sur son Yacht par Albert I Prince Souverain de Monaco 30: 1–96. 10.5962/bhl.title.59328

[B227] Sars M (1857) Bidrag til Kundskaben om Middelhavets Littoral-Fauna, Reisebemærkninger fra Italien. Johan Dahls-Forlag, Christiania, 55 pp.

[B228] Schmidt H (1972) Die Nesselkapseln der Anthozoen und ihre Bedeutung fur die phylogenetische Systematik. Helgoländer Wissenschaftliche Meeresuntersuchungen 23: 422–458. 10.1007/BF01625294

[B229] Schmidt H (1974) On evolution in the Anthozoa. Proceedings of the Second International Coral Reef Symposium 1: 533–560.

[B230] Schückel U Siegfried E Kröncke I (2010) Temporal variability of three different macrofauna communities in the northern North Sea. Estuarine, Coastal and Shelf Science 89: 1–11. 10.1016/j.ecss.2010.04.006

[B231] Sciberras M Hinz H Bennell JD Jenkins SR Hawkins SH Kaiser MJ (2013) Benthic community response to a scallop dredging closure within a dynamic seabed habitat. Marine Ecology Progress Series 480: 83–98. 10.3354/meps10198

[B232] Sebens KP (1998) Anthozoa: Actiniaria, Zoanthidea, Corallimorpharia, and Ceriantharia. In: Pearce JB (Ed.) , Marine Flora and Fauna of the Eastern United States.National Marine Fisheries Service, Seattle, 1–67.

[B233] Shepard AN Theroux RB Cooper RA Uzmann JR (1986) Ecology of Ceriantharia (Coelenterata, Anthozoa) of the northwest Atlantic from Cape Hatteras to Nova Scotia. Fishery Bulletin 84: 625–646.

[B234] Silveira FL Morandini AC (2011) Checklist dos Cnidaria do Estado de São Paulo, Brasil. Biota Neotropica 11: 1–10. 10.1590/S1676-06032011000500016

[B235] Song J-I (1986) First report of a tube anemone, *Cerianthus filiformis* Carlgren, from Korean waters, including comparison of cnidae in adults and planulae. Korean Journal of Systematic Zoology 2: 79–87. 10.1080/12265071.1998.9647407

[B236] Song J-I (1998) Taxonomy of *Cerianthus filiformis* (Ceriantharia, Anthozoa) and its phoronid associate, *Phoronis australis* in Korea. Korean Journal of Biological Science 2: 195–201.

[B237] Song J-I (2000) Cnidaria 2: Anthozoa. Korea Inst. Daejeon, 332 pp.

[B238] Song J-I Lee IS (1998) Fauna of the anthozoans from adjacent waters of Geojedo Island in Korea. Korean Journal of Systematic Zoology 14: 229–242.

[B239] Spallanzani L (1784) Diversi animali nuovi. Memorie della Società Italiana di Verona 2: 627–629.

[B240] Spier D Stampar SN Prantoni AL (2012) New record of the endangered cerianthid *Ceriantheomorphe brasiliensis* (Cnidaria: Hexacorallia) in Paranaguá Bay, southern Brazil. Marine Biodiversity Records 5: e119. 10.1017/S1755267212001078

[B241] Stampar SN Silveira FLD (2006) Espécies sem glamour: lista de animais brasileiros ameaçados de extinção negligencia invertebrados. Scientific American Brazil (52): 10–11.

[B242] Stampar SN Emig C Morandini AC Kodja G Balboni AP Silveira FL (2010) Is there any risk in a symbiotic species associating with an endangered one? A case of a phoronid worm growing on a *Ceriantheomorphe* tube. Cahiers de Biologie Marine 51: 207–211. Available from: http://cbm-online.sb-roscoff.fr/pdf/cb51-2-205-211.pdf

[B243] Stampar SN Maronna MM Vermeij MJA Silveira FLD Morandini AC (2012) Evolutionary diversification of banded tube-dwelling anemones (Cnidaria; Ceriantharia; *Isarachnanthus*) in the Atlantic Ocean. PLoS ONE 7: e4109. 10.1371/journal.pone.0041091 PMC3397977 22815928

[B244] Stampar SN Maronna MM Kitahara M V. Reimer JD Morandini AC (2014a) Fast-Evolving mitochondrial DNA in Ceriantharia: A reflection of Hexacorallia paraphyly? PLoS ONE 9: e86612. 10.1371/journal.pone.0086612 PMC3903554 24475157

[B245] Stampar SN Morandini AC Da Silveira FL (2014b) A new species of *Pachycerianthus* (Cnidaria, Anthozoa, Ceriantharia) from tropical southwestern Atlantic. Zootaxa 3827: 343–354. 10.11646/zootaxa.3827.3.4 25081164

[B246] Stampar SN Beneti JS Acuña FH Morandini AC (2015a) Ultrastructure and tube formation in Ceriantharia (Cnidaria, Anthozoa). Zoologischer Anzeiger 254: 67–71. 10.1016/j.jcz.2014.11.004

[B247] Stampar SN Morandini AC Branco LC Da Silveira FL Migotto AE (2015b) Drifting in the oceans: *Isarachnanthus nocturnus* (Cnidaria, Ceriantharia, Arachnactidae), an anthozoan with an extended planktonic stage. Marine Biology 162: 2161–2169. 10.1007/s00227-015-2747-0

[B248] Stampar SN Scarabino F Pastorino G Morandini AC (2015c) A new species of tube-dwelling anemone (Cnidaria, Anthozoa, Ceriantharia, *Ceriantheopsis*) from the Warm Temperate South-western Atlantic. Journal of the Marine Biological Association of the United Kingdom: 96(7): 1475–1481. 10.1017/S0025315415001745

[B249] Stampar SN Maronna MM Kitahara M V Reimer JD Beneti JS Morandini AC (2016) Ceriantharia in Current Systematics: Life Cycles, Morphology and Genetics. In: Goffredo S Dubinsky Z (Eds) , The Cnidaria, Past, Present and Future: The world of Medusa and her sisters.Springer International Publishing, Cham, 61–72. 10.1007/978-3-319-31305-4_5

[B250] Stampar SN González-Muñoz R Morandini AC (2017) *Botruanthus mexicanus* (Cnidaria: Ceriantharia), a new species of tube-dwelling anemone from the Gulf of Mexico. Marine Biodiversity 47: 113–118. 10.1007/s12526-016-0521-2

[B251] Stampar SN Morandini AC (2017) Occurrence of *Isarachnanthus* (Cnidaria: Anthozoa: Ceriantharia) at Ascension Island: A test of hypothesis. Journal of the Marine Biological Association of the United Kingdom 97: 689–693. 10.1017/S0025315414000423

[B252] Stampar SN El Didi SO Paulay G Berumen ML (2018) A new species of *Arachnanthus* from the Red Sea (Cnidaria, Ceriantharia). ZooKeys 748: 1–10. 10.3897/zookeys.748.22914 PMC5904562 29674909

[B253] Stampar SN Broe MB Macrander J Reitzel AM Brugler MR Daly M (2019) Linear mitochondrial genome in Anthozoa (Cnidaria): A case study in Ceriantharia Scientific Reports 9: 6094. 10.1038/s41598-019-42621-z PMC6465557 30988357

[B254] Stephenson TA (1928) The British Sea Anemones. Volume I. The Ray Society, London, 148 pp.

[B255] Strain EMA Allcock AL Goodwin CE Maggs CA Picton BE Roberts D (2012) The long-term impacts of fisheries on epifaunal assemblage function and structure, in a Special Area of Conservation. Journal of Sea Research 67: 58–68. 10.1016/j.seares.2011.10.001

[B256] Tarasov VG Propp M V Propp LN Zhirmunsky A V Namsakakv BB Gorlenko VM Starynin DA (1990) Shallow water gasohydrothermal vents of Ushishir Volcano and the ecosystem of Kraternaya Bight (the Kurile Islands). Marine Ecology 11: 1–23. 10.1111/j.1439-0485.1990.tb00225.x

[B257] Teissier G (1950) Inventaire de la faune marine de Roscoff Cnidaires et Cténaires. Station Biologique de Roscoff, Roscoff, 42 pp.

[B258] Tiffon Y Hugon JS (1977) Ultrastructural localization of acid and alkaline phosphatases in sterile septae of the Anthozoa*Pachycerianthus fimbriatus*. Histochemistry 54: 289–297. 10.1007/BF00508272 23364

[B259] Torelli B (1932) Nuova specie di *Cerianthus* del Golfo di Napoli (*Cerianthus viridis*). Pubblicazioni della Stazione Zoologica di Napoli 12: 1–15.

[B260] Torelli B (1961) Un *Cerianthus* del Golfo di Napoli: *C. bicyclus* n. sp. (Anthozoa). Pubblicazioni della Stazione Zoologica di Napoli 32: 17–28.

[B261] Torelli B (1963) Due larve di Ceriantharia del Golfo di Napoli (Anthozoa). Pubblicazioni della Stazione Zoologica di Napoli 33: 169–177.

[B262] Torrey HB Kleeberger FL (1909) Contributions from the laboratory of the Marine Biological Association of San Diego. XXVII. Three species of *Cerianthus* from southern California. University of California Publications in Zoology 6: 115–125.

[B263] Tur JM Godall P (1982) Consideraciones preliminares sobre la ecología de los antozoos del litoral sur de la Costa Brava. Oecologia Aquatica: 175–183.

[B264] Uchida H (1979) Cerianthids (Anthozoa, Coelenterata) from Kii Region, Middle Japan. Memoirs of the National Science Museum 12: 185–199.

[B265] Uchida H Soyama I (2001) Sea Anemones in Japanese Waters. TBS, Tokyo, 157 pp.

[B266] Vafidis D Koukouras A (1998) Antipatharia, Ceriantharia, and Zoantharia (Hexacorallia, Anthozoa) of the Aegean Sea with a check list of the Mediterranean and Black Sea species. Annales de l’Institute Oceanographique 74: 115–126.

[B267] van Beneden E (1897) Les Anthozoaires de la “Plankton-Expedition” (Die Anthozoen der Plankton-Expedition). Ergebnisse der Plankton-Expedition der Humboldt-Stiftung 2K: 1–222.

[B268] van Beneden E (1924) Travaux posthumes d’ Edouard van Beneden sur les cérianthaires collationnés par Paul Cerfontaine. Archives de Biologie (hors série): 1–242.

[B269] van Soest RWM (1979) A catalogue of the coelenterate type specimens of the Zoological Museum of Amsterdam. IV. Gorgonacea, Actiniaria, Scleractinia. Beaufortia 29: 81–126.

[B270] Verrill AE (1864a) List of the polyps and corals sent by the Museum of Comparative Zoölogy to other institutions in exchange, with annotations. Bulletin of the Museum of Comparative Zoology 1: 29–60.

[B271] Verrill AE (1864b) Revision of the *Polypi* of the eastern coast of the United States. Memoirs of the Boston Society of Natural History 1: 1–45. 10.5962/bhl.title.78052

[B272] Verrill AE (1865) Classification of polyps (Extract condensed from a Synopsis of the Polypi of the North Pacific Exploring Expedition, under Captains Ringgold and Rodgers, U.S.N.). Proceedings of the Essex Institute 4: 145–152. 10.1080/00222936508679407

[B273] Verrill AE (1868) Synopsis of the polyps and corals of the North Pacific Exploring Expedition under Commodore C. Ringgold and Captain John Rodgers, USN, from 1853–1856. Collected by Dr. Wm. Stimpson, naturalist to the Expedition. With descriptions of some additional species from the west coast of North America. Part III. Madreporaria. Proceedings of the Essex Institute 5: 315–330.

[B274] Verrill AE (1870) Synopsis of the polyps and corals of the North Pacific Exploring Expedition, under Commodore C. Ringgold and Capt. John Rodgers, USN, from 1853 to 1856. Collected by Dr. Wm. Stimpson, Naturalist to the Expedition. Part IV. Actiniaria. Proceedings of the Essex Institute 5: 51–104.

[B275] Verrill AE (1872) On Radiata from the coast of North Carolina. American Journal of Science and Arts 3: 432–438. 10.2475/ajs.s3-3.18.432

[B276] Verrill AE (1873a) Explorations of Casco Bay by the U.S. Fish Commission, in 1873. Proceedings of the American Association for the Advancement of Science 22: 340–395.

[B277] Verrill AE (1873b) Results of recent dredging expeditions on the coast of New England. No. 3. American Journal of Science and Arts 5: 1–15. 10.2475/ajs.s3-5.26.98

[B278] Verrill AE (1873c) Results of recent dredging expeditions on the coast of New England. No. 3. American Journal of Science and Arts 5: 435–441. 10.2475/ajs.s3-5.26.98

[B279] Verrill AE (1874) Results of recent dredging expeditions on the coast of New England. No. 6. American Journal of Science and Arts 7: 405–413. 10.2475/ajs.s3-7.40.405

[B280] Verrill AE (1879) Preliminary check-list of the marine Invertebrata of the Atlantic Coast, from Cape Cod to the Gulf of St. Lawrence. Tuttle, Morehouse & Taylor, Printers, New Haven, 32 pp.

[B281] Verrill AE (1901) Additions to the fauna of the Bermudas from the Yale Expedition of 1901, with notes on other species. Transactions of the Connecticut Academy of Arts and Sciences 11: 15–62.

[B282] Verrill AE (1922) The Actiniaria of the Canadian Arctic Expeditions, with notes on interesting species from Hudson Bay and other Canadian localities. Report on the Canadian Arctic Expedition 1913–1918 8: 89–164.

[B283] Vieira LM Stampar SN (2014) A new *Fenestrulina* (Bryozoa, Cheilostomata) commensal with tube-dwelling anemones (Cnidaria, Ceriantharia) in the tropical southwestern Atlantic. Zootaxa 3780: 365–374. 10.11646/zootaxa.3780.2.8 24871841

[B284] Walton CL (1908) Actiniae collected by the S.S. “Huxley” in the North Sea during the summer of 1907. Journal of the Marine Biological Association of the United Kingdom 8: 215–226. 10.1017/S0025315400072532

[B285] Wassilieff A (1908) Japanische Actinien. Abhandlungen des Mathematischen-Physikalischen Institutes der Kaiserlichen Bayerischen Akademie der Wissenschaften Suppl 1: 1–49.

[B286] Widersten B (1976) Ceriantharia, Zoanthidea, Corallimorpharia, and Actiniaria from the continental shelf and slope off the eastern coast of the United States. Fishery Bulletin 74: 857–878.

[B287] Wiedenmann J Ivanchenko S Oswald F Nienhaus GU (2004) Identification of GFP-like Proteins in Nonbioluminescent, Azooxanthellate Anthozoa Opens New Perspectives for Bioprospecting. Marine Biotechnology 6: 270–277. 10.1007/s10126-004-3006-4 15136917

[B288] Wieking G Kröncke I (2005) Is benthic trophic structure affected by food quality? The Dogger Bank example. Marine Biology 146: 387–400. 10.1007/s00227-004-1443-2

[B289] Williams G (1954) Fauna of Strangford Lough and neighbouring coasts. Proceedings of the Royal Irish Academy 56: 29–123.

[B290] Williams RM (1981) A sea anemone, *Edwardsia meridionalis* sp. nov., from Antarctica and a preliminary revision of the genus *Edwardsia* De Quatrefages, 1841 (Coelenterata: Actiniaria). Records of the Australian Museum 33: 325–360. 10.3853/j.0067-1975.33.1981.271

[B291] Wirtz P Ocaña O Molodtsova TN (2003) Actiniaria and Ceriantharia of the Azores (CnidariaAnthozoa). Helgoland Marine Research 57: 114–117. 10.1007/s10152-003-0146-2

[B292] WoRMS (2020) Anthozoa http://marinespecies.org/aphia.php?p=taxdetails&id=1292

